# Efficacy and safety of erythromycin and metoclopramide, alone or in combination, for successful feeding in critically Ill patients: a systematic review and network meta-analysis

**DOI:** 10.3389/fnut.2026.1834838

**Published:** 2026-09-09

**Authors:** Ming-wei Liu, Xiao-yu Yang, Shan-lan Yang, Ni Ma, Shu-ji Gao, Ye-zi Yang, Yan Liu

**Affiliations:** 1 Department of Emergency, Dali Bai Autonomous Prefecture People’s Hospital, Dali, China; 2 Department of Oncology, Dali Bai Autonomous Prefecture People’s Hospital, Dali, China; 3 Department of Pharmacy, Dali Bai Autonomous Prefecture People’s Hospital, Dali, China; 4 Department of Emergency, The First Affiliated Hospital of Kunming Medical University, Kunming, China; 5 Department of Gastroenterology, Lincang City People’s Hospital, Lincang, Yunnan, China

**Keywords:** critical care, critical illness, enteral nutrition, erythromycin, meta-analysis, metoclopramide

## Abstract

**Background:**

The comparative effectiveness and safety of erythromycin, metoclopramide, and their combination for enteral nutrition in critically ill patients remain uncertain. This network meta-analysis (NMA) synthesized available evidence.

**Methods:**

A systematic search of seven electronic databases (Embase, PubMed, Web of Science, Cochrane Library, CNKI, Wanfang, VIP) from inception to February 6, 2026, was conducted with no language restrictions. Standard Cochrane methods were used for study selection, data extraction, and risk of bias assessment. A frequentist NMA was performed using STATA 18.0 with the mvmeta package. Effect estimates were calculated as odds ratios (OR) for binary outcomes and mean differences (MD) for gastric residual volume (GRV), each with 95% confidence intervals (CI). Random-effects models were used throughout. The surface under the cumulative ranking curve (SUCRA) was used to rank interventions. Sensitivity analyses excluded two pediatric studies. Certainty of evidence was assessed using the CINeMA framework (GRADE for NMA).

**Results:**

Seventeen studies (2,654 patients) were included. For feeding success (5 trials), combination therapy was most effective [SUCRA 100.0%; OR vs. erythromycin 3.03 (1.90–4.83); vs. metoclopramide 4.89 (3.01–7.93)]. For postpyloric intubation success (10 trials), no significant pairwise differences were found; combination therapy ranked highest (SUCRA 76.4%) but with very wide confidence intervals. GRV change (3 trials) showed no significant differences. For any reported AEs (12 trials), placebo (SUCRA 80.0%) and metoclopramide (65.6%) had the most favorable safety profiles, while combination therapy ranked worst (7.5%). The certainty of evidence was moderate for feeding success, low to moderate for AEs, and very low for postpyloric intubation success and GRV.

**Conclusion:**

Combination therapy may be the most effective for feeding success, though based on small trials. Metoclopramide had a better safety profile. No significant differences were observed for postpyloric intubation success or GRV. These preliminary findings require confirmation in larger, well-designed trials.

**Systematic review registration:**

https://www.crd.york.ac.uk/prospero/, identifier CRD420251247900.

## Background

Current guidelines from the American Society for Parenteral and Enteral Nutrition (ASPEN) recommend early enteral nutrition (EN) as the primary approach for critically ill patients with stable hemodynamics, normal gastrointestinal function, and inadequate oral intake (1). Research has shown that initiating EN within 24 h may be associated with reduced gastrointestinal permeability, infection complications, mortality, ICU stays, and overall treatment costs (2, 3). However, gastric motility dysfunction in these patients often leads to feeding intolerance (FI), with rates ranging from 43 to 63% (4–9). Critically ill children also frequently experience gastric dysmotility, with delayed gastric emptying reported in up to 50% of pediatric ICU patients (10). The ASPEN guidelines define EN intolerance based on clinical signs and symptoms observed during daily monitoring, such as abdominal distension, emesis, diarrhea, or unexplained abdominal pain, and discourage routine GRV monitoring in the absence of these signs (1). In contrast, the ESPEN guidelines provide specific quantitative criteria, including gastric residuals >500 mL, worsening abdominal distention or pain, nausea or vomiting ≥3 times/day, diarrhea > 3 times/day or > 500 mL/day, intra-abdominal pressure > 20 mmHg, or the need to reduce or stop EN due to persistent gastrointestinal symptoms for more than 24 h (11). Among these patients, 15–30% have gastric emptying abnormalities (12–18). FI and gastric motility dysfunction may hinder caloric intake, are associated with increase aspiration risk due to mechanical ventilation (19–23), prolong ICU stays, and increase mortality risk (12, 13, 24). Several guidelines suggest prioritizing postgastric-pyloric feeding for patients with FI, reflux, or aspiration risks (11, 25), but clinical success rates are low, which may be largely attributable to gastric motility issues and gastric emptying abnormalities (26).

Gastric emptying abnormalities in critically ill patients stem from multiple factors, including dysfunction of the gastric antrum, which may disrupts normal gastric contraction propagation to the duodenum (4–9, 27). Gastrointestinal function is assessed by monitoring the gastric residual volume (GRV) and performing physical abdominal examinations (22, 23, 28). Treatment options for increasing the GRV include modifying the EN plan, using prokinetic drugs, switching from intragastric to postpyloric EN, or starting parenteral nutrition. Prokinetic drugs are often considered a preferred therapy (19–23, 28–30) and are essential for increasing the success rate of postpyloric feeding tube placement (31).

Approximately 22% of critically ill patients use prokinetic drugs to enhance enteral nutrition absorption, with erythromycin and metoclopramide being the most common (18, 32). Erythromycin, a macrolide antibiotic, promotes gastric emptying by activating the motilin receptor, whereas metoclopramide improves gastrointestinal motility by blocking dopamine receptors (33, 34). Studies have shown that erythromycin reduces the GRV by 75%, metoclopramide by 47%, and combined treatment can reduce the GRV by 92% (35). However, these agents are not without risks. Erythromycin has been associated with dose-related gastrointestinal adverse events (e.g., nausea, vomiting, diarrhea) and QTc interval prolongation, which may increase the risk of cardiac arrhythmias (36). Metoclopramide can cause extrapyramidal symptoms, including acute dystonic reactions and akathisia, particularly in younger patients and at higher doses (37). Previous studies have reported conflicting opinions on the effectiveness of monotherapy versus combination regimens (38, 39), indicating that efficacy may vary depending on patient condition and administration method. In addition, prokinetic agents are also used as a rescue strategy to facilitate postpyloric feeding tube placement. OuYang et al. reported 57.5% success for erythromycin, 50.3% for metoclopramide, and 76% for combined treatment in postpyloric catheterization (40), suggesting a potential synergistic effect. Child studies also have shown that administering metoclopramide intravenously may improve the success rate of bedside blind peristaltic gastrostomy tube insertion in critically ill children (41).

In clinical practice, treatment strategies are often multimodal, and patient responses vary, highlighting the need for a comprehensive evidence synthesis approach. Prior meta-analyses on prokinetics in this population have notable limitations: one focused solely on erythromycin monotherapy using pre-2013 data (42), while another confirmed erythromycin’s prokinetic effect but included only a single placebo-controlled trial for metoclopramide and did not utilize network meta-analysis (43). Neither analysis could rank multiple interventions or estimate the comparative effectiveness of combination therapy versus monotherapy in the absence of head-to-head trials. Our network meta-analysis addresses this gap by synthesizing direct and indirect evidence for erythromycin, metoclopramide, their combination, placebo, and blank control within a unified framework. Furthermore, although gastric residual volume (GRV) is widely used to assess feeding intolerance, its correlation with gastric emptying and aspiration risk is poor, and recent guidelines (1, 44) no longer recommend routine GRV monitoring. This controversy further supports the use of a composite endpoint—successful feeding—rather than isolated GRV thresholds. Accordingly, this study employs network meta-analysis to evaluate the efficacy and safety of the three prokinetic regimens, providing additional evidence to inform clinical decision-making.

## Methods

This systematic review and meta-analysis was performed following the PRISMA 2020 guidelines (45). The protocol number that we registered on the PROSPERO is CRD420251247900.

### Literature search

Based on the PICOS framework, we established inclusion and exclusion criteria and identified relevant articles that met these criteria. To identify studies on erythromycin and/or metoclopramide for treating abnormal gastric emptying in critically ill patients, we conducted a comprehensive search of the following seven databases from their inception to February 6, 2026: Embase, PubMed, Web of Science, the Cochrane Library, China National Knowledge Infrastructure (CNKI), Wanfang Database, and China Science and Technology Journal Database (VIP Database). The search strategy combined MeSH terms and free text, incorporating keywords such as “*Erythromycin,” “Metoclopramide,” and “Critical Illness.”* The specific search strategies used in each database and their corresponding results are presented in Supplementary material. Two researchers independently conducted the literature search and screening. Any disagreements regarding the included studies were resolved through discussion.

### Eligibility criteria

Eligibility criteria for study selection in this review:

(1) Population: Patients admitted to the intensive care unit (ICU) and currently receiving or scheduled to receive enteral nutrition. There were no restrictions on the types of critical illnesses or intubation methods for enteral nutrition.

(2) Intervention and Control: Studies comparing any of the following interventions were eligible for inclusion: erythromycin monotherapy, metoclopramide monotherapy, combination therapy with erythromycin and metoclopramide, placebo, and blank control;

(3) Results: At least one of the primary or secondary outcomes was reported in the included studies.

The primary outcome was successful feeding rate, defined as a gastric residual volume (GRV) decreased below the safety threshold used in each original study, combined with the absence of clinical symptoms of feeding intolerance.

Secondary outcomes included: (1) postpyloric intubation success rate, defined as radiologically confirmed tube tip position beyond the pylorus; (2) change in GRV before and after treatment, calculated as the difference between post-treatment and baseline values; and (3) any reported adverse events (any AEs), defined as any unfavorable medical occurrence documented in the original trials, irrespective of attribution to the study drug. (4) No restrictions were imposed regarding study design, publication language or publication status.

The exclusion criteria were as follows:

(1) Meetings, reviews, case reports, and protocols were excluded;

(2) Duplicate published studies or datasets.

### Data extraction

The data extraction process was carried out by two independent investigators who operated separately. Any discrepancies were resolved through discussion. The investigators extracted both basic characteristic data and outcome data from studies that met the predefined inclusion criteria. The basic characteristic data included the following elements: author(s) of the article, year of publication, research design, research location, type of severe condition, sample size, average age, proportion of male participants, types of prokinetic drugs (erythromycin monotherapy, metoclopramide monotherapy, or combination therapy), single dosage, and research findings. The efficiency and safety outcomes included the successful feeding rate, postpyloric intubation success rate and any AEs.

### Risk of bias assessment

The quality of the included randomized controlled trials (RCTs) was assessed via the “Cochrane Risk of Bias Assessment Tool Version 2 (RoB2) (46), whereas nonrandomized studies were evaluated via the “Risk of Bias in Nonrandomized Studies of Interventions (ROBINS-I)” tool (47). Two independent assessors performed the quality assessments separately, addressing any disagreements through discussion. The RoB2 tool encompasses five domains, with risks of bias in each domain and overall classified as “low risk,” “some concerns,” or “high risk.” The ROBINS-I tool assesses the quality of nonrandomized intervention studies across seven aspects, with the results categorized into five levels: low risk, moderate risk, serious risk, critical risk, and no relevant information.

### Statistical analysis

Within the frequentist framework (43), all analyses and visualizations were conducted in STATA (version 18.0) using the mvmeta package and its associated tools. Effect estimates for the dichotomous outcomes—feeding success, post-pyloric tube success, and any AEs rate—were calculated using odds ratios (ORs), which are presented with 95% confidence intervals (CIs). For the continuous outcome, GRV change, mean difference (MD) was used because the original studies reported the outcome using the same units and measurement methods; we extracted the change from baseline to the final measurement time point (typically day 7 or study endpoint), calculated as post-treatment minus baseline when only separate values were reported, and imputed the variance using a correlation coefficient of 0.5.

Heterogeneity was assessed using *I*^2^ (with values > 50% indicating high heterogeneity) and the between-study standard deviation τ (and τ^2^), estimated from a random-effects model. To evaluate the consistency assumption, we performed a global inconsistency test (design-by-treatment interaction, χ^2^ test) and local node-splitting analysis for each closed loop. A non-significant *p*-value (*p* > 0.05) was considered supportive of consistency. The efficacy and safety of the various interventions was then ranked using the surface under the cumulative ranking curve (SUCRA). Two-sided *P* < 0.05 was considered to indicate statistical significance. Small-study effects and potential publication bias were evaluated using comparison-specific funnel plots. To assess the potential impact of including two pediatric trials (41, 48), a sensitivity analysis by repeating the network meta-analysis after excluding these studies were performed.

To further illustrate the incidence of any AEs across different gastric motility regimens, we qualitatively summarized the types and numbers of any AEs reported in previous monotherapy and combination therapy studies.

### Transitivity assumption and assessment of effect modifiers

The validity of network meta-analysis relies on the transitivity assumption, which requires that the distribution of potential effect modifiers is comparable across different treatment comparisons within the network (49). To evaluate the plausibility of transitivity, we compared the following study-level characteristics that may act as effect modifiers across pairwise comparisons: (1) patient population (adults vs. children), (2) severity of illness (APACHE II or equivalent scores), (3) use of concomitant medications (sedatives, opioids, vasopressors), (4) erythromycin dosage, (5) definition of outcome measures (e.g., GRV thresholds for feeding success, radiological criteria for postpyloric placement), and (6) intervention protocols (route, timing, and duration of administration). The distributions of these characteristics were tabulated and visually inspected for systematic differences across comparisons. Formal inconsistency tests were also performed for each outcome (reported in the respective sections) to provide statistical support for the consistency assumption.

### GRADE assessment

The certainty of evidence for each network meta-analysis estimate was assessed using the Confidence in Network Meta-Analysis framework (CINeMA), which operationalizes the GRADE approach for network meta-analysis. Five domains were evaluated: risk of bias, inconsistency, indirectness, imprecision, and publication bias. No upgrading factors—such as large effect, dose-response gradient, or plausible residual confounding that would reduce the effect—were applied, as the data did not support them. For each comparison, the overall certainty was rated as high, moderate, low, or very low.

## Results

### Study selection

**FIGURE 1:**
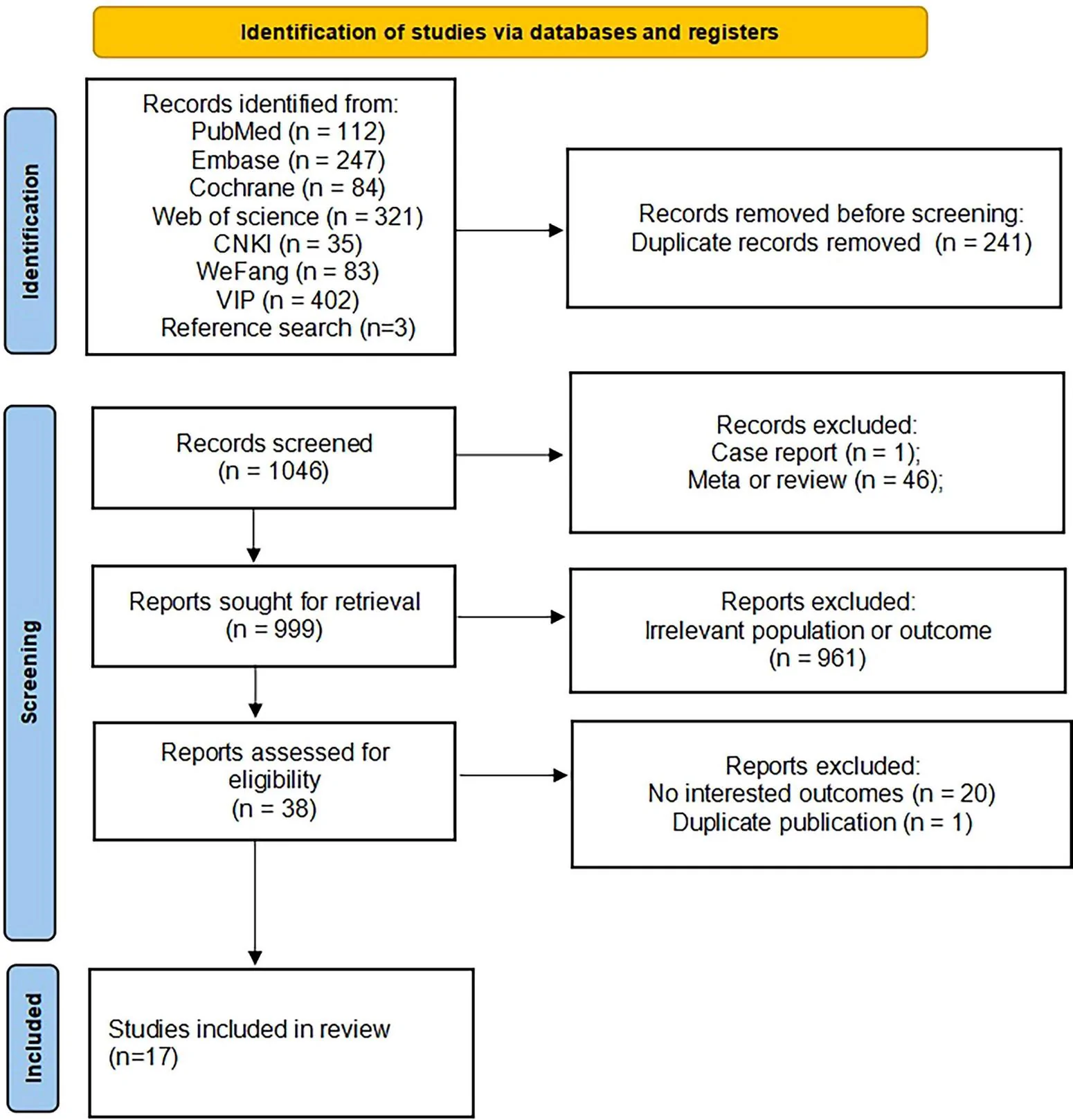
Flowchart of study selection.

A total of 1,284 records were identified through database searching (PubMed: 112; Embase: 247; Cochrane Library: 84; Web of Science: 321; CNKI: 35; WanFang: 83; VIP: 402), and an additional 3 records were identified through reference searching, giving a total of 1,287 records. After the removal of 241 duplicate records, 1,046 records remained for title and abstract screening. Based on the screening, 1,008 records were excluded for the following reasons: case report (*n* = 1), meta-analysis or review article (*n* = 46), and irrelevant population or outcomes (*n* = 961). The full texts of the remaining 38 articles were retrieved for detailed assessment. Of these, 20 articles were excluded for not reporting the outcomes of interest, and 1 was excluded as a duplicate publication. Ultimately, 17 studies (35, 38–41, 48, 50–60) met the inclusion criteria and were included in this systematic review and network meta-analysis. The study selection process was illustrated in Figure 1. To ensure the authenticity of the data and the reproducibility of the research results, we included English versions of the abstracts from the Chinese studies, along with traceable literature website information, in Supplementary Table 1.

### Study characteristics and assessment of the transitivity assumption

The publication years of the 17 included studies ranged from 1995 to 2025. The studies were conducted in several countries, including China (*n* = 7), the United States (*n* = 6), Australia (*n* = 2), Thailand (*n* = 1), and India (*n* = 1). Most studies (*n* = 16) were RCTs, while one study was a retrospective trial. The sample sizes varied considerably across the studies, ranging from 20 to 777 participants, with a total of 2,654 critically ill patients included across all the studies. The proportion of male participants ranged from 51.4 to 84.3%, with several studies reporting more than 60% male representation. The mean age of the participants ranged from approximately 1.8–69.1 years, with most studies focusing on adult populations, except for those by Gharpure et al. (48) and Ketsuwan et al. (41), which included pediatric cohorts. The interventions evaluated included erythromycin, metoclopramide, combination therapy (erythromycin + metoclopramide), placebo, or a blank control. Dosages varied across studies, with erythromycin dosages ranging from 70 mg to 500 mg, metoclopramide from 10 mg to 20 mg, and combination therapy from 0.25 mg/kg to 250 mg + 10 mg. The study characteristics are summarized in Table 1.

**TABLE 1:** Characteristics of the included studies.

Study	Country	Study design	Sample size	Male%	Age (mean ± SD)	Intervention	Outcomes
Chen et al. (51)	China	RCT	120	63.3%	69.1 ± 16.6	ERY/metoclopramide	Postpyloric intubation success
Dickerson et al. (55)	United States	Retrospective trial	127	75.6%	40.4 ± 15.9	Metoclopramide/combine	Efficacy rates, AEs
Feng (52)	China	RCT	68	57.35%	52.12 ± 6.10	ERY/combine	Postpyloric intubation success
Gharpure et al. (48)	United States	RCT	74	51.4%	3.5 (0.1–16)/1.8 (0.1–17)	ERY/placebo	Postpyloric intubation success, AEs
Griffith et al. (56)	United States	RCT	36	69.4%	54.6 ± 3.4/59.7 ± 3.8	ERY/placebo	Postpyloric intubation success
Heiselman et al. (57)	United States	RCT	105	/	/	Metoclopramide/blank control	Postpyloric intubation success
Hu et al. (26)	China	RCT	332	69.0%	57.2 ± 17.3	ERY/metoclopramide	Postpyloric intubation success, AEs
Ketsuwan et al. (41)	Thailand	RCT	82	53.7%	21 Months/17 months	Metoclopramide/placebo	Postpyloric intubation success
Liang et al. (58)	China	RCT	777	65.5%	67	ERY/metoclopramide/combine	Postpyloric intubation success, AEs
Liu (62)	China	RCT	120	67.5%	66.15 ± 2.25	ERY/metoclopramide/parallel combine	Successful rate of feeding, AEs^&^
Lu et al. (60)	China	RCT	152	/	/	ERY/metoclopramide/combine	Successful rate of feeding
Maclaren (20)	United States	RCT	20	70.0%	49.55	ERY/metoclopramide	Tmax, Cmax, C60, AUC0-60, GRV change
Nguyen et al. (53)	Australia	RCT	61	70.7%	52.1 ± 4.1	ERY/combine	Successful rate of feeding, GRV change, AEs
Nguyen et al. (35)	Australia	RCT	90	73.3%	49.0 ± 13.4	ERY/metoclopramide/combine	Successful rate of feeding, GRV change
Paz et al. (59)	United States	RCT	83	53.0%	60.4 ± 18.3	ERY/metoclopramide/placebo	Postpyloric intubation success
Vijayaraghavan et al. (54)	India	RCT	83	84.3%	46.1 ± 12.4	ERY/metoclopramide/placebo	Successful rate of feeding
Zhang et al. (50)	China	RCT	243 (324)*	54.6%	59.2	ERY/metoclopramide/combine	Successful rate of feeding

RCT, randomized controlled trial; ERY, erythromycin; MET, metoclopramide; COMB, combination therapy; AEs, adverse events; GRV, gastric residual volume. Data are mean ± SD or median (range). “/” = not reported. Blank control = no prokinetic intervention. Dickerson 2009 was retrospective; all others were RCTs. Pediatric studies: Gharpure 2001 and Ketsuwan 2021. Safety outcomes: any reported AEs (see Supplementary Table 4 for details).

*: Total enrolled 324 patients including a naloxone arm not part of the network; only 243 patients in the three intervention groups (metoclopramide, erythromycin, combination) were included in the NMA.

^&^:Combination group data were used only for qualitative safety description and were not included in the feeding success network meta-analysis as a parallel treatment node.

Based on these characteristics, this study further assessed the transitivity assumption of the network meta-analysis by comparing the distribution of key potential effect modifiers across treatment comparisons, including patient age (pediatric vs. adult), erythromycin dosage, disease severity (APACHE II or equivalent scores), and outcome definitions (e.g., GRV thresholds for feeding success, radiological criteria for postpyloric placement). These distributions are detailed in Supplementary Table 2. While some variability was observed—particularly in age and dosage—no systematic imbalance was identified that would obviously favor one treatment comparison over another. Formal inconsistency tests performed for each outcome (see the respective sections) were all non-significant (*p* > 0.05), providing statistical support for the consistency assumption. However, given the limited number of studies, the potential impact of residual intransitivity cannot be fully excluded.

### Study quality

**FIGURE 2:**
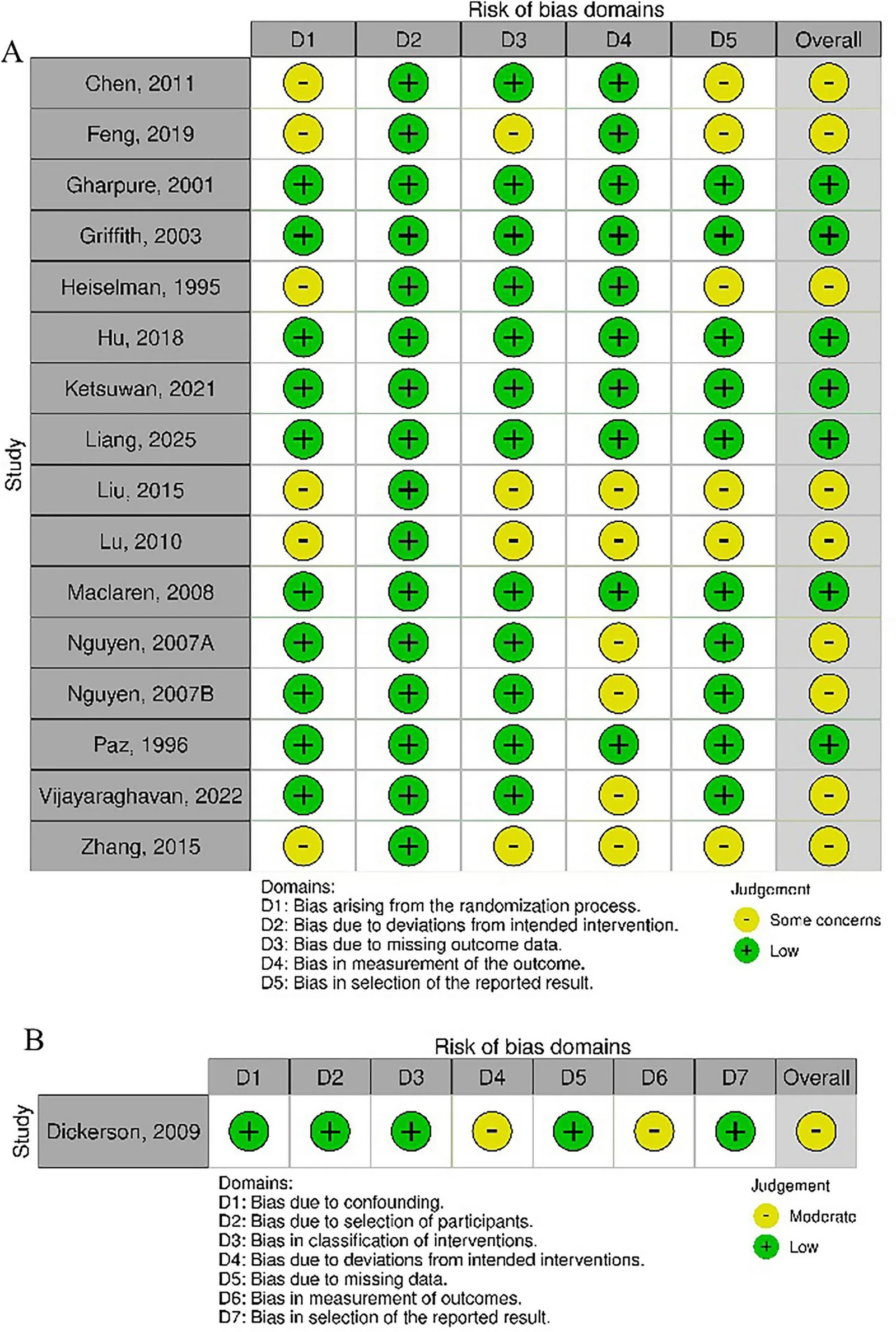
Quality evaluation of **(A)** RCTs by RoB2; **(B)** non-RCT intervention studies by ROBINS-I.

In the present systematic review and network meta-analysis, the methodological quality of the 17 included studies (16 RCTs and 1 nonrandomized interventional study) varied. According to the ROB2 assessment, most international multicenter RCTs (35, 40, 41, 48, 53, 56, 58, 59) demonstrated a low risk of bias across domains, including randomization, intervention adherence, outcome measurement, and reporting, indicating high overall quality. In contrast, several single-center studies (39, 50–52, 57, 60) showed “some concerns,” primarily due to insufficient reporting of randomization procedures, incomplete outcome data, and potential selective reporting (Figure 2A).

The nonrandomized interventional study (55), assessed with the ROBINS-I, was judged to have a moderate risk of bias (Figure 2B). This was primarily due to outcome measurement, as gastric residual volume was determined by manual aspiration, a method prone to operator variability and subjective thresholds, and confounding, since the concomitant use of sedatives, muscle relaxants, barbiturates, and insulin infusion could independently affect gastric motility and thereby interfere with the observed intervention effects.

### Successful feeding rates

**FIGURE 3:**
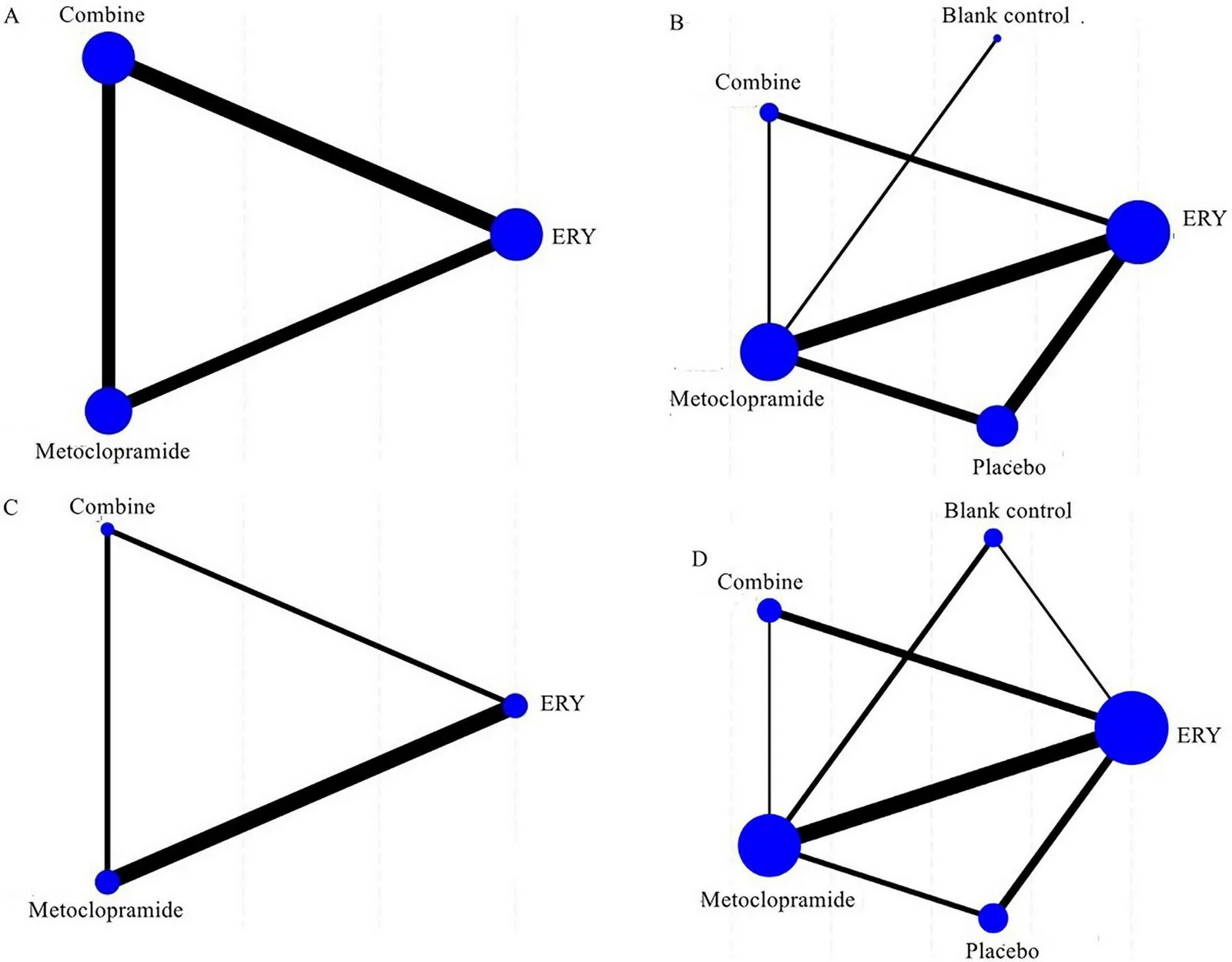
Network analysis of eligible comparisons for **(A)** successful feeding rate; **(B)** postpyloric intubation success; **(C)** GRV; **(D)** TRAE.

This study employed a consistency network meta-analysis model to synthesize evidence from five trials (35, 39, 50, 53, 60) evaluating the efficacy of erythromycin, metoclopramide, and their combination in achieving successful feeding among 747 critically ill patients. The network was fully connected (Figure 3A), and the test for inconsistency revealed no significant differences between direct and indirect evidence (χ^2^ = 0.03, *p* = 0.87), supporting the validity of the model. The estimated between-study standard deviation (τ) was 8.6 × 10^–13^, with τ^2^ = 7.4 × 10 ^–25^, indicating negligible heterogeneity. Contribution analysis indicated that the three direct comparisons contributed relatively evenly to the overall network (ERY vs. combine: 34.9%, ERY vs. metoclopramide: 32.7%, combine vs. metoclopramide: 32.4%), suggesting no major imbalance in direct contributions (Supplementary Figure 1).

Pairwise comparisons demonstrated that combination therapy was significantly superior to erythromycin (OR = 3.03; 95% CI: 1.90–4.83; *p* < 0.001) and metoclopramide (OR = 4.89; 95% CI: 3.01–7.93; *p* < 0.001), whereas erythromycin was significantly more effective than metoclopramide was (OR = 1.61; 95% CI: 1.06–2.45; *p* = 0.025) (Table 2A).

**TABLE 2:** League tables for (a) Successful feeding rate; (b) Postpyloric intubation success; (c) GRV; (d) Treatment-related adverse effect.

a. Successful feeding rate (OR, 95% CI)				
**Combine**	3.03 (1.90, 4.83)		0.62 (0.41, 0.94)	
0.33 (0.21, 0.53)	**Erythromycin**		0.20 (0.13, 0.33)	
1.61 (1.06, 2.45)	4.89 (3.01, 7.93)		**Metoclopramide**	
**b. Postpyloric intubation success (OR, 95% CI)**				
**ERY**	0.71 (0.05, 10.91)	1.69 (0.31, 9.10)	1.00 (0.31, 3.24)	0.41 (0.10, 1.69)
1.41 (0.09, 21.69)	**Blankcontrol**	2.38 (0.11, 51.54)	1.41 (0.12, 16.64)	0.58 (0.03, 11.14)
0.59 (0.11, 3.19)	0.42 (0.02, 9.09)	**Combine**	0.59 (0.09, 3.71)	0.24 (0.03, 2.07)
1.00 (0.31, 3.24)	0.71 (0.06, 8.36)	1.69 (0.27, 10.57)	**Metoclopramide**	0.41 (0.08, 2.08)
2.42 (0.59, 9.90)	1.72 (0.09, 32.78)	4.09 (0.48, 34.57)	2.42 (0.48, 12.19)	**Placebo**
**c. GRV (MD, 95% CI)**				
**ERY**	−11.28 (−206.68, 184.12)		−65.67 (−192.97, 61.63)	
11.28 (−184.12, 206.68)	**Combine**		−54.39 (−249.86, 141.08)	
65.67 (−61.63, 192.97)	54.39 (−141.08, 249.86)		**Metoclopramide**	
**d. Any reported adverse events (OR, 95% CI)**				
**ERY**	0.53 (0.05, 6.20)	1.45 (0.99, 2.14)	0.70 (0.51, 0.96)	0.38 (0.08, 1.81)
1.88 (0.16, 21.94)	**Blankcontrol**	2.73 (0.23, 32.51)	1.31 (0.11, 15.20)	0.72 (0.04, 13.11)
0.69 (0.47, 1.01)	0.37 (0.03, 4.36)	**Combine**	0.48 (0.31, 0.74)	0.26 (0.05, 1.30)
1.43 (1.04, 1.97)	0.76 (0.07, 8.81)	2.08 (1.36, 3.18)	**Metoclopramide**	0.55 (0.12, 2.63)
2.60 (0.55, 12.23)	1.38 (0.08, 25.08)	3.78 (0.77, 18.52)	1.82 (0.38, 8.68)	**Placebo**

For each league table, estimates in the lower triangle represent the odds ratio (or mean difference) for the row treatment compared with the column treatment; estimates in the upper triangle represent the column treatment compared with the row treatment. For odds ratios, values < 1 favor the column treatment, values > 1 favor the row treatment.

Ranking analysis further confirmed that combination therapy had the highest probability of being the most effective (SUCRA = 100.0%, PrBest = 100.0%), followed by erythromycin (SUCRA = 49.4%), and metoclopramide ranked lowest (SUCRA = 0.6%) (Table 3 and Supplementary Figure 2).

**TABLE 3:** Surface under the cumulative ranking curve (SUCRA) probabilities (%) and mean rank of interventions.

Treatments	Successful feeding rate		Postpyloric intubation success		GRV		Any reported adverse events	
	SUCRA	MeanRank	SUCRA	MeanRank	SUCRA	MeanRank	SUCRA	MeanRank
Combine	100.0 %	1.0	76.4 %	1.9	58.5%	1.8	7.5%	4.7
Erythromycin	49.4%	2.0	56.5%	2.7	69.3%	1.6	35.3%	3.6
Metoclopramide	0.6%	3.0	56.0%	2.8	22.3%	2.6	65.6%	2.4
Placebo	/	/	43.0%	3.3	/	/	80.0%	1.8
Blank control	/	/	18.0%	4.3	/	/	61.6%	2.5

Funnel plot inspection did not reveal obvious asymmetry; however, the limited number of studies restricts the power to detect publication bias (Supplementary Figure 3).

### Postpyloric intubation success rates

Based on a consistency network meta-analysis model, this study synthesizes evidence from 10 trials (40, 41, 48, 51, 52, 54, 56–59) evaluating erythromycin, metoclopramide, their combination, a blank control, and a placebo for achieving postpyloric intubation success among 1,760 critically ill patients. The network was fully connected (Figure 3B), and the estimated between-study standard deviation (τ) was 1.20 in the consistency model (τ^2^ = 1.44), indicating substantial heterogeneity. The inconsistency model did not show statistically significant superiority over the consistency model (χ^2^ = 1.82, *p* = 0.18), indicating that the consistency assumption is acceptable. However, the substantial heterogeneity among the studies (SD = 1.20) suggests caution in interpreting the results. Contribution analysis revealed that the main direct comparisons contributed to the entire network as follows: ERY vs. metoclopramide (30.3%), ERY vs. placebo (17.7%), blank control vs. metoclopramide (22.7%), combine vs. metoclopramide (21.6%), and metoclopramide vs. placebo (5.1%) (Supplementary Figure 4).

Pairwise comparisons revealed that combination therapy did not significantly outperform erythromycin (OR = 1.69, 95% CI: 0.31–9.10), metoclopramide (OR = 1.69, 95% CI: 0.27–10.57), or the blank control (OR = 2.38, 95% CI: 0.11–51.5). Similarly, erythromycin and metoclopramide did not significantly differ (OR = 1.00, 95% CI: 0.31–3.24). Placebo consistently demonstrated the lowest success rates across comparisons, although the differences did not reach statistical significance (Table 2B).

Ranking analysis indicated that combination therapy had the highest probability of being the most effective (SUCRA = 76.4%, PrBest = 52.1%), followed by erythromycin (SUCRA = 56.5%), metoclopramide (SUCRA = 56.0%), the placebo (SUCRA = 43.0%), and the blank control (SUCRA = 18.0%) (Table 3 and Supplementary Figure 5).

Funnel plot inspection did not reveal obvious asymmetry; however, the limited number of studies restricts the power to detect publication bias (Supplementary Figure 6).

To assess the potential impact of including two pediatric trials (41, 48), we performed a sensitivity analysis by repeating the network meta-analysis after excluding these studies. The ranking results based on the remaining eight adult-only trials are presented in Supplementary Table 3. Combination therapy remained the highest-ranked intervention (SUCRA = 77.2%, PrBest = 54.8%), followed by erythromycin (SUCRA = 52.1%, PrBest = 5.0%) and metoclopramide (SUCRA = 46.6%, PrBest = 11.0%). The overall ranking pattern was consistent with the primary analysis, suggesting that inclusion of the pediatric studies did not materially alter the relative treatment hierarchy. However, confidence intervals for pairwise comparisons remained very wide, reflecting persistent imprecision.

### Changes in the gastric residual volume (GRV) before and after treatment

This study employed a consistency network meta-analysis model to evaluate changes in the GRV before and after treatment across three interventions: erythromycin, metoclopramide, and their combination. A total of four studies (35, 38, 50, 53) with 495 patients were included, and the network was fully connected (Figure 3C). Overall heterogeneity was substantial, with an estimated between-study standard deviation (τ) of 107.2 (τ^2^ = 11,492), although the inconsistency test was not significant (χ^2^ = 0.36, *p* = 0.55). Contribution analysis indicated that direct comparisons contributed relatively evenly to the overall network (ERY vs. combine: 45.6%, ERY vs. metoclopramide: 9.1%, combine vs. metoclopramide: 45.3%), suggesting that the contribution pattern was relatively balanced (Supplementary Figure 7).

Pairwise comparisons revealed no statistically significant differences among the three treatments. Specifically, combination therapy versus erythromycin therapy yielded a mean difference of –11.28 mL (95% CI: –206.68 to 184.12; *p* = 0.91), and metoclopramide therapy versus erythromycin therapy resulted in a mean difference of –65.67 mL (95% CI: –192.97 to 61.63; *p* = 0.31), and metoclopramide versus combination therapy resulted in a mean difference of –54.39 mL (95% CI: –249.86 to 141.08; *p* = 0.31) (Table 2C).

Ranking analysis suggested that erythromycin had the highest probability of being the most effective (SUCRA = 69.3%, PrBest = 48.9%), followed by combination therapy (SUCRA = 58.5%, PrBest = 43.9%), while metoclopramide ranked lowest (SUCRA = 22.3%, PrBest = 7.2%) (Table 3 and Supplementary Figure 8).

Funnel plot inspection did not reveal obvious asymmetry; however, the very small number of studies (*n* = 3) limits the power to detect publication bias (Supplementary Figure 9).

### Any reported adverse events (AEs)

This study employed a consistency network meta-analysis model to evaluate any reported AEs across five interventions—erythromycin, blank control, combination therapy, metoclopramide, and placebo—based on 12 included studies (*n* = 1,878) (38–41, 48, 51–54, 57–59). The network was fully connected (Figure 3D). The test for inconsistency showed no significant disagreement between direct and indirect evidence (χ^2^ = 0.66, *p* = 0.9986), supporting the validity of the consistency model. The estimated between-study standard deviation (τ) was 8.3 × 10^–10^, with τ^2^ ≈ 6.9 × 10^−1^9^^, indicating negligible heterogeneity. Contribution analysis demonstrated that direct comparisons contributed to the entire network as follows: ERY vs. blank control (8.5%), ERY vs. combine (5.7%), ERY vs. metoclopramide (27.3%), ERY vs. placebo (16.9%), blank control vs. metoclopramide (14.9%), combine vs. metoclopramide (20.0%), and metoclopramide vs. placebo (6.7%) (Supplementary Figure 10).

Pairwise comparisons revealed that compared with erythromycin, metoclopramide was associated with significantly fewer any reported AEs (OR = 0.70; 95% CI: 0.51–0.96; *p* = 0.028). The combination therapy also showed a numerically higher AE rate than erythromycin, although not statistically significant (OR = 1.45; 95% CI: 0.99–2.14; *p* = 0.059). Blank control (OR = 0.53; 95% CI: 0.05–6.20) and placebo (OR = 0.38; 95% CI: 0.08–1.81) did not differ significantly from erythromycin. Comparisons between active treatments showed that combination therapy was associated with significantly more AEs than metoclopramide (OR = 2.08; 95% CI: 1.36–3.18; *p* = 0.001) (Table 2D).

Ranking analysis indicated that placebo had the highest probability of being the safest (SUCRA = 80.0%, PrBest = 52.3%, mean rank = 1.8), followed by metoclopramide (SUCRA = 65.6%, PrBest = 9.4%, mean rank = 2.4) and blank control (SUCRA = 61.6%, PrBest = 38.2%, mean rank = 2.5). Erythromycin (SUCRA = 35.3%, mean rank = 3.6) and combination therapy (SUCRA = 7.5%, mean rank = 4.7) ranked worst (Table 3 and Supplementary Figure 11).

Funnel plot inspection did not reveal obvious asymmetry; however, the limited number of studies restricts the power to detect publication bias (Supplementary Figure 12).

To assess the potential impact of including two pediatric trials (41, 48), we repeated the network meta-analysis after excluding these studies. The ranking pattern remained consistent: metoclopramide (SUCRA = 74.0%, PrBest = 23.0%) and blank control (SUCRA = 66.7%, PrBest = 46.6%) continued to show the most favorable safety profiles, while combination therapy (SUCRA = 14.6%) and erythromycin (SUCRA = 43.8%) ranked lower (Supplementary Table 4). These results support the robustness of the primary findings.

### Systematic review of specific adverse events

Owing to the presence of diverse definitions, incomplete reporting, and inadequate individual-level overlap data pertaining to specific adverse events, a reliable quantitative synthesis could not be achieved. Consequently, we provide the following qualitative summary as Supplementary Information.

A total of 13 studies (38–41, 48, 51–53, 55–59) reported data on specific adverse events across the four intervention groups (erythromycin, metoclopramide, combination therapy, placebo or blank control); studies with no mention of safety outcomes (35, 50, 54, 60) were excluded. The reported AEs were predominantly digestive symptoms (nausea, vomiting, diarrhea, abdominal pain), followed by elevated liver enzymes (aspartate aminotransferase, alanine aminotransferase, alkaline phosphatase). Other events included allergic reactions, arrhythmias, and procedure-related complications (e.g., nasal mucosa bleeding, airway misplacement). No study used a standardized adverse event scale (e.g., CTCAE) or systematically monitored pre-defined events such as QTc prolongation or extrapyramidal symptoms.

Digestive symptoms were the most commonly reported AEs across all intervention groups, particularly diarrhea. Liver enzyme elevations were more frequently observed in erythromycin-containing regimens, especially combination therapy. Notably, Nguyen 2007A (53) reported the highest incidence of any AE (54.0%) in the combination group, all of which were diarrhea. Procedure-related complications (e.g., nasal bleeding, airway misplacement) occurred in a small proportion of patients (generally < 5%) and were not associated with drug treatment. Because original studies did not provide individual-level overlap data (e.g., a patient with both diarrhea and nausea would be counted separately for each symptom), the absolute numbers for specific symptom categories (e.g., digestive) may overestimate the actual number of affected patients. Therefore, the primary safety analysis in this study uses any reported AE (composite endpoint) rather than symptom-specific events. Detailed information is presented in Supplementary Table 5.

### Certainty of evidence

The certainty of evidence was moderate for feeding success and for the comparison of metoclopramide versus erythromycin in any reported adverse events (as the 95% CI no longer crossed the null). For most other comparisons of any reported adverse events, the certainty was low, downgraded mainly for imprecision and risk of bias. The certainty was very low for postpyloric intubation success and gastric residual volume, downgraded for risk of bias, imprecision (wide confidence intervals), and, for postpyloric intubation success, also inconsistency and indirectness. Detailed GRADE evidence tables are provided in Supplementary Table 6.

## Discussion

This network meta-analysis evaluated the efficacy and safety of erythromycin, metoclopramide, and their combination for enteral nutrition in critically ill patients. For feeding success, combination therapy was most effective, but this finding derives from a small network of five trials with very low heterogeneity; the ceiling SUCRA and PrBest values (100.0%) should be interpreted with caution. For postpyloric intubation success, combination therapy ranked highest, but pairwise differences were non-significant, and wide confidence intervals and substantial heterogeneity (SD = 1.20) indicate considerable uncertainty. Erythromycin ranked highest for reducing gastric residual volume, but this analysis included only three studies and is exploratory. Regarding safety, metoclopramide and placebo had the most favorable profiles, whereas combination therapy was associated with the highest adverse event rates. Overall, the evidence base is small and the results are preliminary.

With respect to the feeding success rate, our analysis indicated that combined therapy outperforms erythromycin, and erythromycin in turn outperforms metoclopramide. These findings highlight the synergistic effects of the combined therapy. Erythromycin and metoclopramide act on the gastrointestinal system through different mechanisms—erythromycin primarily enhances proximal gastric motility, whereas metoclopramide regulates gastric sphincter function and overall peristalsis. When used together, these drugs can produce synergistic effects at different targets, improving the GRV and feeding success rate and thus enhancing patients’ ability to tolerate enteral feeding (61). Liu (62) evaluated combination therapy as a rescue strategy in patients who had failed monotherapy and observed that the effect could persist for up to 7 days after switching to the combination. However, this finding applies to the salvage setting and does not directly inform the durability of upfront combination therapy or its ability to prevent tachyphylaxis (39). However, studies on combined therapy are relatively rare, and there is considerable variability in the regimens used, necessitating further high-quality research to confirm this preliminary conclusion. The extremely high SUCRA and PrBest values (100.0%) for combination therapy in this outcome should be interpreted with caution, as they arise from a small network of five trials with very low heterogeneity and may not fully reflect real-world variability. Meanwhile, recent trials have evaluated mosapride and prucalopride for feeding intolerance in critically ill patients. Mosapride (5-HT4 agonist) reduced GRV and increased enteral volume compared with metoclopramide (63). Prucalopride also reduced GRV and improved caloric intake (64). Both showed tolerable safety. However, head-to-head comparisons with erythromycin and metoclopramide are lacking, and no studies have examined synergistic combinations. Future network meta-analyses incorporating these agents are needed to define their therapeutic role.

For postpyloric intubation success, no statistically significant differences were observed among the three treatments. Although combination therapy ranked highest in the exploratory SUCRA analysis (76.4%) and some original studies have suggested potential benefits (40, 51), the pairwise comparisons in this NMA did not demonstrate superiority of any regimen. In neurocritical care patients, the spiral-type postpyloric placement method, combined with prokinetic drugs, helps establish a postpyloric feeding pathway, avoiding the risks associated with patient transport for tube insertion or bedside endoscopic placement (31, 40). Head-to-head comparisons of only erythromycin and combination therapy revealed that the combination treatment achieved 91.18% catheterization success, whereas the success rate was 70.59% with erythromycin alone (52). Although few head-to-head studies are available, some single-arm studies suggest that erythromycin and metoclopramide may affect the success rate of gastric tube placement to varying degrees, with erythromycin showing potential in small sample studies (57, 65). Consequently, given the current limited sample size, drawing a definitive conclusion remains challenging.

For GRV, no statistically significant differences were observed among the three treatments. The SUCRA rankings suggested a probabilistic ordering (erythromycin ranked highest, followed by combination therapy, then metoclopramide), but these exploratory rankings should not be interpreted as evidence of superior efficacy given the very wide confidence intervals and the small number of studies (*n* = 3). Although previous studies have proposed mechanistic rationales for erythromycin as a motilin agonist (66–68), and some individual trials have suggested numerical improvements with erythromycin or metoclopramide (38, 50, 60), the present network meta-analysis does not provide sufficient evidence to conclude that any one regimen is superior to another for reducing GRV. Larger, well-designed trials are needed to clarify the comparative effectiveness among these interventions.

In this analysis, metoclopramide and placebo exhibited the most favorable safety profiles, whereas erythromycin and combination therapy were associated with higher adverse event rates. This ordering aligns with the known pharmacology of the two agents. Erythromycin, even at low prokinetic doses (70–200 mg), can cause dose-related gastrointestinal disturbances including nausea, vomiting, abdominal pain and diarrhea; higher doses ( ≥ 500 mg) act primarily as antibiotics and may worsen these effects. Moreover, erythromycin has been shown to mildly but significantly prolong the QTc interval, warranting ECG monitoring in at-risk patients (36). Metoclopramide carries a well-recognized risk of extrapyramidal symptoms, with acute dystonic reactions occurring in approximately 1 in 500 adults treated with standard doses, typically within the first 24–48 h (37). In the short-term ICU setting, such events are rarely reported, but akathisia and other movement disorders remain a consideration. Previous meta-analyses of prokinetics as a drug class did not find a significant increase in diarrhea compared with placebo (69), and overall adverse events were not statistically different from control (69, 70). However, combination therapy with erythromycin and metoclopramide has been associated with a very high incidence of diarrhea (up to 49%), which is more common than with either agent alone; this diarrhea is not linked to Clostridium difficile infection and resolves after drug withdrawal (53). Thus, the increased gastrointestinal burden of combination therapy likely reflects additive prokinetic and gastrointestinal effects rather than infectious causes. Both erythromycin and metoclopramide monotherapies are associated with rapid tolerance development via receptor downregulation and desensitization (71, 72), whereas combination therapy may mitigate this by acting on different pharmacological targets (73, 74).

This study involves two main sources of heterogeneity, differences in patient age and intervention dosage. Erythromycin doses varied widely, and its prokinetic effect is dose-dependent. Specifically, 70 mg and 200 mg are equally effective in accelerating gastric emptying in critically ill adults (34), while higher doses (≥ 500 mg) may primarily function as antibiotics and increase gastrointestinal adverse events, such as nausea and cramping, potentially counteracting feeding success (75). Consequently, the average effect estimates we report should be interpreted as representing a range of doses rather than a single optimal regimen. Pharmacokinetic and pharmacodynamic profiles also differ significantly between children and adults. In children, both 1 mg/kg and 3 mg/kg of erythromycin produce similar prokinetic effects, although the higher dose is associated with more side effects (76). Additionally, metoclopramide clearance in children is weight-dependent (77), and pediatric gastroparesis differs substantially from adult disease in terms of etiology and treatment response (78). These differences may violate the transitivity assumption when mixing pediatric and adult studies. However, due to the small number of trials, formal dose-stratified or population-specific network meta-analyses were not feasible. Therefore, our effect estimates represent averages across heterogeneous doses and populations, and caution is warranted when applying them to specific dosing protocols or to children.

### Limitations

This study has several limitations. First, the evidence base is small for all outcomes, with only five trials for feeding success and three for GRV, limiting statistical power and precision. Second, substantial clinical heterogeneity existed across studies, including wide variations in erythromycin dosage (70–500 mg), patient age (1.8–69.1 years, mixing children and adults), outcome definitions (e.g., different GRV thresholds and radiological criteria), and intervention protocols (route, timing, duration). Formal dose-stratified or pediatric-only subgroup analyses were not feasible due to sparse data. Third, one non-randomized study, Dickerson et al. (55) was included to maintain network connectivity; although a sensitivity analysis excluding it yielded similar findings, its moderate risk of bias may affect internal validity. Fourth, the ceiling SUCRA and PrBest values (100.0%) for feeding success arise from a very small network with negligible heterogeneity and should not be interpreted as absolute guarantees of superiority. Fifth, safety outcomes were limited by the lack of standardized adverse event definitions (e.g., no systematic monitoring of QTc prolongation or extrapyramidal symptoms) and the use of a composite “any AE” endpoint, which mixes events of different clinical relevance. Sixth, transitivity and consistency assumptions may not be fully satisfied given the heterogeneity; although inconsistency tests were non-significant, the limited study numbers reduce their reliability. Seventh, most trials reported only short-term outcomes, leaving long-term safety and resistance concerns unexplored. Therefore, future research should focus on adequately powered, multicenter randomized trials with standardized protocols and outcome definitions, longer follow-up for safety and resistance monitoring, and, where possible, individual patient data meta-analyses to better adjust for confounders and explore effect modifiers.

## Conclusion

In this network meta-analysis, combination therapy was the most effective for feeding success, though based on a small number of trials. Metoclopramide had a more favorable safety profile than erythromycin-containing regimens, while other safety comparisons were of low certainty. No statistically significant differences were found for postpyloric intubation success or gastric residual volume, with very low certainty in the evidence. These findings need validation in larger, high-quality clinical trials before definitive recommendations can be made.

## References

[1] McClave SA Taylor BE Martindale RG Warren MM Johnson DR Braunschweig C et al. Guidelines for the Provision and Assessment of Nutrition Support Therapy in the Adult Critically Ill Patient: Society of Critical Care Medicine (SCCM) and American Society for Parenteral and Enteral Nutrition (A.S.P.E.N.). *JPEN J Parenter Enteral Nutr.* (2016) 40:159–211. 10.1177/0148607115621863 26773077

[2] Doig GS Simpson F. Early enteral nutrition in the critically ill: do we need more evidence or better evidence? *Curr Opin Crit Care.* (2006) 12:126–30. 10.1097/01.ccx.0000216579.34310.84 16543788

[3] Artinian V Krayem H DiGiovine B. Effects of early enteral feeding on the outcome of critically ill mechanically ventilated medical patients. *Chest.* (2006) 129:960–7. 10.1378/chest.129.4.960 16608945

[4] Mutlu GM Mutlu EA Factor P. GI complications in patients receiving mechanical ventilation. *Chest.* (2001) 119:1222–41. 10.1378/chest.119.4.1222 11296191

[5] Fruhwald S Holzer P Metzler H. Intestinal motility disturbances in intensive care patients pathogenesis and clinical impact. *Intensive Care Med.* (2007) 33:36–44. 10.1007/s00134-006-0452-7 17115132

[6] Chapman MJ Nguyen NQ Fraser RJ. Gastrointestinal motility and prokinetics in the critically ill. *Curr Opin Crit Care.* (2007) 13:187–94. 10.1097/MCC.0b013e3280523a88 17327741

[7] Dive A Moulart M Jonard P Jamart J Mahieu P. Gastroduodenal motility in mechanically ventilated critically ill patients: a manometric study. *Crit Care Med.* (1994) 22:441–7. 10.1097/00003246-199403000-00014 8124995

[8] Ritz MA Fraser R Edwards N Di Matteo AC Chapman M Butler R et al. Delayed gastric emptying in ventilated critically ill patients: measurement by 13 C-octanoic acid breath test. *Crit Care Med.* (2001) 29:1744–9. 10.1097/00003246-200109000-00015 11546976

[9] Deane A Chapman MJ Fraser RJ Bryant LK Burgstad C Nguyen NQ. Mechanisms underlying feed intolerance in the critically ill: implications for treatment. *World J Gastroenterol.* (2007) 13:3909–17. 10.3748/wjg.v13.i29.3909 17663503 PMC4171161

[10] Martinez EE Douglas K Nurko S Mehta NM. Gastric dysmotility in critically Ill children: pathophysiology, diagnosis, and management. *Pediatr Crit Care Med.* (2015) 16:828–36. 10.1097/pcc.0000000000000493 26218259 PMC4635032

[11] Singer P Blaser AR Berger MM Alhazzani W Calder PC Casaer MP et al. ESPEN guideline on clinical nutrition in the intensive care unit. *Clin Nutr.* (2019) 38:48–79. 10.1016/j.clnu.2018.08.037 30348463

[12] Montejo JC. Enteral nutrition-related gastrointestinal complications in critically ill patients: a multicenter study. The Nutritional and Metabolic Working Group of the Spanish Society of Intensive Care Medicine and Coronary Units. *Crit Care Med.* (1999) 27:1447–53. 10.1097/00003246-199908000-00006 10470748

[13] Chang RW Jacobs S Lee B. Gastrointestinal dysfunction among intensive care unit patients. *Crit Care Med.* (1987) 15:909–14. 10.1097/00003246-198710000-00003 3115678

[14] Heyland D Cook DJ Winder B Brylowski L Van deMark H Guyatt G. Enteral nutrition in the critically ill patient: a prospective survey. *Crit Care Med.* (1995) 23:1055–60. 10.1097/00003246-199506000-00010 7774216

[15] Binnekade JM Tepaske R Bruynzeel P Mathus-Vliegen EM de Hann RJ. Daily enteral feeding practice on the ICU: attainment of goals and interfering factors. *Crit Care.* (2005) 9:R218–25. 10.1186/cc3504 15987393 PMC1175883

[16] Adam S Batson SA. study of problems associated with the delivery of enteral feed in critically ill patients in five ICUs in the UK. *Intensive Care Med.* (1997) 23:261–6. 10.1007/s001340050326 9083227

[17] McClave SA Sexton LK Spain DA Adams JL Owens NA Sullins MB et al. Enteral tube feeding in the intensive care unit: factors impeding adequate delivery. *Crit Care Med.* (1999) 27:1252–6. 10.1097/00003246-199907000-00003 10446815

[18] Heyland DK Dhaliwal R Day A Jain M Drover J. Validation of the Canadian clinical practice guidelines for nutrition support in mechanically ventilated, critically ill adult patients: results of a prospective observational study. *Crit Care Med.* (2004) 32:2260–6. 10.1097/01.ccm.0000145581.54571.32 15640639

[19] Kazi N Mobarhan S. Enteral feeding associated gastroesophageal reflux and aspiration pneumonia: a review. *Nutr Rev.* (1996) 54:324–8. 10.1111/j.1753-4887.1996.tb03796.x 9063023

[20] MacLaren R. Intolerance to intragastric enteral nutrition in critically ill patients: complications and management. *Pharmacotherapy.* (2000) 20:1486–98. 10.1592/phco.20.19.1486.34853 11130221

[21] Metheny NA Schallom ME Edwards SJ. Effect of gastrointestinal motility and feeding tube site on aspiration risk in critically ill patients: a review. *Heart Lung.* (2004) 33:131–45. 10.1016/j.hrtlng.2004.02.001 15136773

[22] Dodek P Keenan S Cook D Heyland D Jacka M Hand L et al. Evidence-based clinical practice guideline for the prevention of ventilator-associated pneumonia. *Ann Intern Med.* (2004) 141:305–13. 10.7326/0003-4819-141-4-200408170-00011 15313747

[23] American Thoracic Society, Infectious Diseases Society of America Guidelines for the management of adults with hospital-acquired, ventilator-associated, and healthcare-associated pneumonia. *Am J Respir Crit Care Med.* (2005) 171:388–416. 10.1164/rccm.200405-644ST 15699079

[24] Mentec H Dupont H Bocchetti M Cani P Ponche F Bleichner G. Upper digestive intolerance during enteral nutrition in critically ill patients: frequency, risk factors, and complications. *Crit Care Med.* (2001) 29:1955–61. 10.1097/00003246-200110000-00018 11588461

[25] Rhodes A Evans LE Alhazzani W Levy MM Antonelli M Ferrer R et al. Surviving sepsis campaign: international guidelines for management of sepsis and septic shock: 2016. *Intensive Care Med.* (2017) 43:304–77. 10.1007/s00134-017-4683-6 28101605

[26] Hu B Lv B Chen C. The choice of a postpyloric tube and the patient’s position in our procedure: a response. *Crit Care.* (2018) 22:127. 10.1186/s13054-018-2036-7 29747674 PMC5946431

[27] Heyland DK Tougas G King D Cook DJ. Impaired gastric emptying in mechanically ventilated, critically ill patients. *Intensive Care Med.* (1996) 22:1339–44. 10.1007/bf01709548 8986483

[28] Metheny NA. Preventing respiratory complications of tube feedings: evidence-based practice. *Am J Crit Care.* (2006) 15:360–9. 10.4037/ajcc2006.15.4.360 16823013

[29] Davies AR Bellomo R. Establishment of enteral nutrition: prokinetic agents and small bowel feeding tubes. *Curr Opin Crit Care.* (2004) 10:156–61. 10.1097/00075198-200404000-00013 15075727

[30] Booth CM Heyland DK Paterson WG. Gastrointestinal promotility drugs in the critical care setting: a systematic review of the evidence. *Crit Care Med.* (2002) 30:1429–35. 10.1097/00003246-200207000-00005 12130957

[31] Hu B Ye H Sun C Zhang Y Lao Z Wu F et al. Metoclopramide or domperidone improves post-pyloric placement of spiral nasojejunal tubes in critically ill patients: a prospective, multicenter, open-label, randomized, controlled clinical trial. *Crit Care.* (2015) 19:61. 10.1186/s13054-015-0784-1 25880172 PMC4367875

[32] Heyland DK Schroter-Noppe D Drover JW Jain M Keefe L Dhaliwal R et al. Nutrition support in the critical care setting: current practice in canadian ICUs–opportunities for improvement? *JPEN J Parenter Enteral Nutr.* (2003) 27:74–83. 10.1177/014860710302700174 12549603

[33] Marino LV Kiratu EM French S Nathoo N. To determine the effect of metoclopramide on gastric emptying in severe head injuries: a prospective, randomized, controlled clinical trial. *Br J Neurosurg.* (2003) 17:24–8. 12779198

[34] Ritz MA Chapman MJ Fraser RJ Finnis ME Butler RN Cmielewski P et al. Erythromycin dose of 70 mg accelerates gastric emptying as effectively as 200 mg in the critically ill. *Intensive Care Med.* (2005) 31:949–54. 10.1007/s00134-005-2663-8 15940460

[35] Nguyen NQ Chapman MJ Fraser RJ Bryant LK Holloway RH. Erythromycin is more effective than metoclopramide in the treatment of feed intolerance in critical illness. *Crit Care Med.* (2007) 35:483–9. 10.1097/01.Ccm.0000253410.36492.E9 17205032

[36] Hawkyard CV Koerner RJ. The use of erythromycin as a gastrointestinal prokinetic agent in adult critical care: benefits versus risks. *J Antimicrob Chemother.* (2007) 59:347–58. 10.1093/jac/dkl537 17289772

[37] Parlak I Atilla R Cicek M Parlak M Erdur B Guryay M et al. Rate of metoclopramide infusion affects the severity and incidence of akathisia. *Emerg Med J.* (2005) 22:621–4. 10.1136/emj.2004.014712 16113179 PMC1726928

[38] MacLaren R Kiser TH Fish DN Wischmeyer PE. Erythromycin vs metoclopramide for facilitating gastric emptying and tolerance to intragastric nutrition in critically ill patients. *JPEN J Parenter Enteral Nutr.* (2008) 32:412–9. 10.1177/0148607108319803 18596312

[39] Zizhuo L. *Eyrthromycin Improves Gastric Emptying in Critically Ill Patients Intolerant of Nasogastric Feeding.* Beijing: Hebei United University (2015). 10.1097/00003246-200007000-00026 10921561

[40] Hu B Ouyang X Lei L Sun C Chi R Guo J et al. Erythromycin versus metoclopramide for post-pyloric spiral nasoenteric tube placement: a randomized non-inferiority trial. *Intensive Care Med.* (2018) 44:2174–82. 10.1007/s00134-018-5466-4 30465070 PMC6280835

[41] Ketsuwan S Tanpowpong P Ruangwattanapaisarn N Phaopant S Suppalarkbunlue N Kooanantkul C et al. Intravenous metoclopramide to improve the success rate of blind bedside post-pyloric placement of feeding tube in critically Ill children: a randomized, double-blind, placebo-controlled study. *Front Pediatr.* (2021) 9:739247. 10.3389/fped.2021.739247 35004534 PMC8727866

[42] Rong L Huilin T Suodi Z Xi Z. Erythromycin for improving enteral nutrition tolerance in adult critical patients: a systematic review and Meta-analysis. *Chin Crit Care Med.* (2014) 26:425–30. 10.3760/cma.j.issn.2095-4352.2014.06.012 24912643

[43] Peng R. The efficacy and safety of administration of prokinetics improve clinical outcomes in critically ill patients is still quite unclear. *Clin Nutr.* (2020) 39:307–9. 10.1016/j.clnu.2019.11.027 31813697

[44] Reignier J Gaillard-Le Roux B Dequin PF Bertoni Maluf VA Bohe J Casaer MP et al. Expert consensus-based clinical practice guidelines for nutritional support in the intensive care unit: the French Intensive Care Society (SRLF) and the French-Speaking Group of Pediatric Emergency Physicians and Intensivists (GFRUP). *Ann Intensive Care.* (2025) 15:99. 10.1186/s13613-025-01509-0 40665004 PMC12263543

[45] Page MJ McKenzie JE Bossuyt PM Boutron I Hoffmann TC Mulrow CD et al. The PRISMA 2020 statement: an updated guideline for reporting systematic reviews. *BMJ.* (2021) 372:n71. 10.1136/bmj.n71 33782057 PMC8005924

[46] Crocker TF Lam N Jordão M Brundle C Prescott M Forster A et al. Risk-of-bias assessment using Cochrane’s revised tool for randomized trials (RoB 2) was useful but challenging and resource-intensive: observations from a systematic review. *J Clin Epidemiol.* (2023) 161:39–45. 10.1016/j.jclinepi.2023.06.015 37364620

[47] Sterne JA Hernán MA Reeves BC Savović J Berkman ND Viswanathan M et al. ROBINS-I: a tool for assessing risk of bias in non-randomised studies of interventions. *BMJ.* (2016) 355:i4919. 10.1136/bmj.i4919 27733354 PMC5062054

[48] Gharpure V Meert KL Sarnaik AP. Efficacy of erythromycin for postpyloric placement of feeding tubes in critically ill children: a randomized, double-blind, placebo controlled study. *JPEN J Parenter Enteral Nutr.* (2001) 25:160–5. 10.1177/0148607101025003160 11334066

[49] Sandau N Aagaard TV Hróbjartsson A Harris IA Brorson S. Transitivity, coherence, and reliability of network meta-analyses comparing proximal humerus fracture treatments: a meta-epidemiological study. *BMC Musculoskelet Disord.* (2024) 25:14. 10.1186/s12891-023-07119-w 38166880 PMC10759380

[50] Zhang L Cao W Li P Sun X Zhang Y He N. Clinical study of naloxone in treatment of gastro-intestinal dysfunction in critically ill patients. *J Crit Care Intern Med.* (2015) 21:24–6. 10.3109/02688690309177968

[51] Chen C Zeng H Wu Y Ye H Sun C Long Y et al. Study on the Enhancement of the Success Rate of Post-Gastric Tube Placement in Spiral Nasoenteric Tube by Erythromycin and Metoclopramide. *Proceedings of the 17th World Conference on Disaster and Emergency Medicine and the 14th National Academic Annual Conference on Emergency Medicine.* Beijing: (2011).

[52] Feng Z. Study on the success rate of manual placement of nasoenteric tube with erythromycin combined with metoclopramide. *Electron J Clin Pharmaceut Liter.* (2019) 6:167. 10.16281/j.cnki.jocml.2019.63.148

[53] Nguyen NQ Chapman M Fraser RJ Bryant LK Burgstad C Holloway RH. Prokinetic therapy for feed intolerance in critical illness: one drug or two? *Crit Care Med.* (2007) 35:2561–7. 10.1097/01.Ccm.0000286397.04815.B1 17828038

[54] Vijayaraghavan R Maiwall R Arora V Choudhary A Benjamin J Aggarwal P et al. Reversal of feed intolerance by prokinetics improves survival in critically Ill cirrhosis patients. *Dig Dis Sci.* (2022) 67:4223–33. 10.1007/s10620-021-07185-x 34392492 PMC8364303

[55] Dickerson RN Mitchell JN Morgan LM Maish GO III Croce MA Minard G et al. Disparate response to metoclopramide therapy for gastric feeding intolerance in trauma patients with and without traumatic brain injury. *JPEN J Parenter Enteral Nutr.* (2009) 33:646–55. 10.1177/0148607109335307 19892902

[56] Griffith DP McNally AT Battey CH Forte SS Cacciatore AM Szeszycki EE et al. Intravenous erythromycin facilitates bedside placement of postpyloric feeding tubes in critically ill adults: a double-blind, randomized, placebo-controlled study. *Crit Care Med.* (2003) 31:39–44. 10.1097/00003246-200301000-00006 12544991

[57] Heiselman DE Hofer T Vidovich RR. Enteral feeding tube placement success with intravenous metoclopramide administration in ICU patients. *Chest.* (1995) 107:1686–8. 10.1378/chest.107.6.1686 7781368

[58] Liang S Huang J He L Chen Y Li C Hu L et al. Erythromycin plus metoclopramide combination therapy compared with erythromycin or metoclopramide monotherapy for postpyloric spiral nasoenteric tube placement: a multicenter, open-label, randomized controlled superiority trial. *Am J Clin Nutr.* (2025) 122:1850–7. 10.1016/j.ajcnut.2025.09.043 41033467

[59] Paz HL Weinar M Sherman MS. Motility agents for the placement of weighted and unweighted feeding tubes in critically ill patients. *Intensive Care Med.* (1996) 22:301–4. 10.1007/bf01700450 8708166

[60] Lu NF Zheng RQ Lin H Yang DG Chen QH Shao J et al. Study of erythromycin and metoclopramide in treatment of feeding intolerance of critically ill patients in intensive care unit. *Zhongguo Wei Zhong Bing Ji Jiu Yi Xue.* (2010) 22:36–9. 10.3760/cma.j.issn.1003-0603.2010.01.015 20092709

[61] Szczupak M Jankowska M Jankowski B Wierzchowska J Kobak J Szczupak P et al. Prokinetic effect of erythromycin in the management of gastroparesis in critically ill patients-our experience and literature review. *Front Med.* (2024) 11:1440992. 10.3389/fmed.2024.1440992 39314225 PMC11416996

[62] Crone V Møller MH Alhazzani W Grønningsæter L Al-Fares A Hästbacka J et al. Preferences on the use of prokinetic agents in adult intensive care unit patients-an international survey. *Acta Anaesthesiol Scand.* (2025) 69:e70045. 10.1111/aas.70045 40275492 PMC12022387

[63] Mohamed Elmokadem E Khaled Abou El Fadl D Bassiouny AM Hanna Samy AE Omar El Said N. Mosapride versus metoclopramide in critically ill patients with enteral feeding intolerance: a randomized, double-blinded comparison. *Drug Des Devel Ther.* (2026) 20:582745. 10.2147/dddt.S582745 41908938 PMC13025770

[64] Elmokadem EM Abou El Fadl DK Eissa N Alnassar NA Bassiouny AM Hanna Samy AE et al. Comparison of enteral prucalopride versus intravenous metoclopramide for feeding intolerance in patients with critical illness: a randomized double-blinded study. *Front Pharmacol.* (2024) 15:1413246. 10.3389/fphar.2024.1413246 39584139 PMC11581857

[65] Whatley K Turner WW Jr. Dey M Leonard J Guthrie M. When does metoclopramide facilitate transpyloric intubation? *JPEN J Parenter Enteral Nutr.* (1984) 8:679–81. 10.1177/0148607184008006679 6441010

[66] Ng E Shah VS. Erythromycin for the prevention and treatment of feeding intolerance in preterm infants. *Cochrane Database Syst Rev.* (2008) 2008:Cd001815. 10.1002/14651858.CD001815.pub2 18646077 PMC12747216

[67] Sanger GJ Westaway SM Barnes AA Macpherson DT Muir AI Jarvie EM et al. GSK962040: a small molecule, selective motilin receptor agonist, effective as a stimulant of human and rabbit gastrointestinal motility. *Neurogastroenterol Motil.* (2009) 21: 657–64, e30–1. 10.1111/j.1365-2982.2008.01270.x 19374732

[68] Deane AM Fraser RJ Chapman MJ. Prokinetic drugs for feed intolerance in critical illness: current and potential therapies. *Crit Care Resusc.* (2009) 11:132–43. 10.1016/S1441-2772(23)01538-7 19485878

[69] Lewis K Alqahtani Z McIntyre L Almenawer S Alshamsi F Rhodes A et al. The efficacy and safety of prokinetic agents in critically ill patients receiving enteral nutrition: a systematic review and meta-analysis of randomized trials. *Crit Care.* (2016) 20:259. 10.1186/s13054-016-1441-z 27527069 PMC4986344

[70] Silva CC Bennett C Saconato H Á N. Metoclopramide for post-pyloric placement of naso-enteral feeding tubes. *Cochrane Database Syst Rev.* (2015) 1:Cd003353. 10.1002/14651858.CD003353.pub2 25564770 PMC7170214

[71] Schade RR Dugas MC Lhotsky DM Gavaler JS Van Thiel DH. Effect of metoclopramide on gastric liquid emptying in patients with diabetic gastroparesis. *Dig Dis Sci.* (1985) 30:10–5. 10.1007/BF01318364 3965270

[72] Janssens J Peeters TL Vantrappen G Tack J Urbain JL De Roo M et al. Improvement of gastric emptying in diabetic gastroparesis by erythromycin. Preliminary studies. *N Engl J Med.* (1990) 322:1028–31. 10.1056/nejm199004123221502 2320062

[73] Thielemans L Depoortere I Perret J Robberecht P Liu Y Thijs T et al. Desensitization of the human motilin receptor by motilides. *J Pharmacol Exp Ther.* (2005) 313:1397–405. 10.1124/jpet.104.081497 15764739

[74] Lamian V Rich A Ma Z Li J Seethala R Gordon D et al. Characterization of agonist-induced motilin receptor trafficking and its implications for tachyphylaxis. *Mol Pharmacol.* (2006) 69:109–18. 10.1124/mol.105.017111 16221873

[75] NuCare Pharmaceuticals Inc *Prescription Drug Information: Erythromycin.* (2026). Available online at: https://rxdruglabels.com/lib/rx/rx-meds/erythromycin/ (accessed May 20, 2026).

[76] Di Lorenzo C Flores AF Tomomasa T Hyman PE. Effect of erythromycin on antroduodenal motility in children with chronic functional gastrointestinal symptoms. *Dig Dis Sci.* (1994) 39:1399–404. 10.1007/bf02088040 8026249

[77] Ge S Mendley SR Gerhart JG Melloni C Hornik CP Sullivan JE et al. Population pharmacokinetics of metoclopramide in infants, children, and adolescents. *Clin Transl Sci*. (2020) 13:1189–98. 10.1111/cts.12803 32324313 PMC7719387

[78] Febo-Rodriguez L Chumpitazi BP Shulman RJ. Childhood gastroparesis is a unique entity in need of further investigation. *Neurogastroenterol Motil.* (2020) 32:e13699. 10.1111/nmo.13699 31407456 PMC7015769

