# Supplementary material for: Efficacy and safety of erythromycin and metoclopramide, alone or in combination, for successful feeding in critically Ill patients: a systematic review and network meta-analysis
Source: Front Nutr. 2026 Sep 9;13:1834838. doi: 10.3389/fnut.2026.1834838 (PMC13597321; doi:10.3389/fnut.2026.1834838)
Supplement: Supplementary file 1 [file Supplementary_file_1.docx]

**Supplementary Figure 1 Contribution analysis for successful feeding rate.**

**Supplementary Figure 2 Cumulative ranking probability plots for successful feeding rate.**

**Supplementary Figure 3 Funnel plot for successful feeding rate.**

**Supplementary Figure 4 Contribution analysis for postpyloric intubation success.**

**Supplementary Figure 5 Cumulative ranking probability plots for postpyloric intubation success.**

**Supplementary Figure 6 Funnel plot for postpyloric intubation success.**

**Supplementary Figure 7 Contribution analysis for GRV.**

**Supplementary Figure 8 Cumulative ranking probability plots for GRV.**

**Supplementary Figure 9 Funnel plot for GRV.**

**Supplementary Figure 10 Contribution analysis for any AEs**

**Supplementary Figure 11 Cumulative ranking probability plots for any AEs.**

**Supplementary Figure 12 Funnel plot for any AEs.**
