# Supplementary material for: Efficacy and safety of erythromycin and metoclopramide, alone or in combination, for successful feeding in critically Ill patients: a systematic review and network meta-analysis
Source: Front Nutr. 2026 Sep 9;13:1834838. doi: 10.3389/fnut.2026.1834838 (PMC13597321; doi:10.3389/fnut.2026.1834838)
Supplement: Supplementary file 2 [file Supplementary_file_2.docx]

**Search strategy of PubMed.**

| NO. | Search Details | Results |
| --- | --- | --- |
| 15 | ((#8) AND (#11)) AND (#14) | 112 |
| 14 | (#12) OR (#13) | 47,067 |
| 13 | ((((((((((((((Nutrition, Enteral) OR (Tube Feeding)) OR (Feeding, Tube)) OR (Enteral Feeding)) OR (Feeding, Enteral)) OR (Gastric Feeding Tubes)) OR (Feeding Tube, Gastric)) OR (Feeding Tubes, Gastric)) OR (Gastric Feeding Tube)) OR (Tube, Gastric Feeding)) OR (Tubes, Gastric Feeding)) OR (Force Feeding)) OR (Feeding, Force)) OR (Feedings, Force)) OR (Force Feedings) | 47,067 |
| 12 | "Enteral Nutrition"[Mesh] | 23,489 |
| 11 | (#9) OR (#10) | 131,357 |
| 10 | (((Critical Illnesses) OR (Illness, Critical)) OR (Illnesses, Critical)) OR (Critically Ill) | 131,357 |
| 9 | "Critical Illness"[Mesh] | 44,749 |
| 8 | ((#3) OR (#6)) OR (#7) | 379,551 |
| 7 | "Placebos"[Mesh] | 36,190 |
| 6 | (#4) OR (#5) | 300,656 |
| 5 | (((((((((((((Metoclopramide) OR (4-Amino-5-chloro-N-(2-(diethylamino)ethyl)-2-methoxybenzamide)) OR (Reglan)) OR (Primperan)) OR (Rimetin)) OR (Maxolon)) OR (Dihydrochloride)) OR (Dihydrochloride, Metoclopramide)) OR (Metoclopramide Hydrochloride)) OR (Hydrochloride, Metoclopramide)) OR (Metoclopramide Monohydrochloride, Monohydrate)) OR (Metoclopramide Monohydrochloride)) OR (Monohydrochloride, Metoclopramide)) OR (Cerucal) | 300,656 |
| 4 | "Metoclopramide"[Mesh] | 5,074 |
| 3 | (#1) OR (#2) | 44,068 |
| 2 | (((((((((((Erythromycin A) OR (Erythromycin Lactate)) OR (Lactate, Erythromycin)) OR (Erythromycin Phosphate)) OR (Phosphate, Erythromycin)) OR (Erythromycin C)) OR (Ilotycin)) OR (Erycette)) OR (Erymax)) OR (T-Stat)) OR (TStat)) OR (T Stat) | 44,068 |
| 1 | "Erythromycin"[Mesh] | 29,270 |

**Search strategy of EMBASE**

| No. | Query | Results |
| --- | --- | --- |
| #15 | #8 AND #11 AND #14 | 247 |
| #14 | #12 OR #13 | 42833 |
| #13 | 'enteral nutrition':ti,ab,kw OR 'nutrition, enteral':ti,ab,kw OR 'tube feeding':ti,ab,kw OR 'feeding, tube':ti,ab,kw OR 'enteral feeding':ti,ab,kw OR 'feeding, enteral':ti,ab,kw OR 'gastric feeding tube':ti,ab,kw OR 'feeding tube, gastric':ti,ab,kw OR 'gastric feeding tubes':ti,ab,kw OR 'feeding tubes, gastric':ti,ab,kw OR 'force feeding':ti,ab,kw OR 'feeding, force':ti,ab,kw OR 'force feedings':ti,ab,kw OR 'feedings, force':ti,ab,kw | 42833 |
| #12 | 'enteral nutrition':ti,ab,kw | 23101 |
| #11 | #9 OR #10 | 130331 |
| #10 | 'critical illnesses':ti,ab,kw OR 'illness, critical':ti,ab,kw OR 'illnesses, critical':ti,ab,kw OR 'critically ill':ti,ab,kw | 113702 |
| #9 | 'critical illness':ti,ab,kw | 28319 |
| #8 | #3 OR #6 OR #7 | 143380 |
| #7 | 'placebos':ti,ab,kw | 5107 |
| #6 | #4 OR #5 | 39077 |
| #5 | 'metoclopramide':ti,ab,kw OR '4-amino-5-chloro-n-(2-(diethylamino)ethyl)-2-methoxybenzamide':ti,ab,kw OR 'reglan':ti,ab,kw OR 'primperan':ti,ab,kw OR 'rimetin':ti,ab,kw OR 'maxolon':ti,ab,kw OR 'dihydrochloride':ti,ab,kw OR 'dihydrochloride, metoclopramide':ti,ab,kw OR 'metoclopramide hydrochloride':ti,ab,kw OR 'hydrochloride, metoclopramide':ti,ab,kw OR 'metoclopramide monohydrochloride, monohydrate':ti,ab,kw OR 'metoclopramide monohydrochloride':ti,ab,kw OR 'monohydrochloride, metoclopramide':ti,ab,kw OR 'cerucal':ti,ab,kw | 8049 |
| #4 | 'metoclopramide' | 31645 |
| #3 | #1 OR #2 | 101404 |
| #2 | 'erythromycin a':ti,ab,kw OR 'erythromycin lactate':ti,ab,kw OR 'lactate, erythromycin':ti,ab,kw OR 'erythromycin phosphate':ti,ab,kw OR 'phosphate, erythromycin':ti,ab,kw OR 'erythromycin c':ti,ab,kw OR 'ilotycin':ti,ab,kw OR 'erycette':ti,ab,kw OR 'erymax':ti,ab,kw OR 't-stat':ti,ab,kw OR 'tstat':ti,ab,kw OR 't stat':ti,ab,kw | 1105 |
| #1 | 'erythromycin'/exp OR 'erythromycin' | 101278 |

**Search strategy of Cochrane Library**

| NO. | Search deatiles | Hits |
| --- | --- | --- |
| #1 | MeSH descriptor: [Erythromycin] explode all trees | 4386 |
| #2 | (Erythromycin A) OR (Erythromycin Lactate) OR (Lactate, Erythromycin) OR (Erythromycin Phosphate) OR (Phosphate, Erythromycin) OR (Erythromycin C) OR (Ilotycin) OR (Erycette) OR (Erymax) OR (T-Stat) OR (TStat) OR (T Stat) | 2567 |
| #3 | #1 OR #2 | 5874 |
| #4 | MeSH descriptor: [Metoclopramide] explode all trees | 1366 |
| #5 | (Metoclopramide) OR (Reglan) OR (Primperan) OR (Rimetin) OR (Maxolon) OR (Dihydrochloride) OR (Dihydrochloride, Metoclopramide) OR (Metoclopramide Hydrochloride) OR (Hydrochloride, Metoclopramide) OR (Metoclopramide Monohydrochloride, Monohydrate) OR (Metoclopramide Monohydrochloride) OR (Monohydrochloride, Metoclopramide) OR (Cerucal) | 927 |
| #6 | #4 OR #5 | 2231 |
| #7 | MeSH descriptor: [Critical Illness] explode all trees | 3947 |
| #8 | (Critical Illnesses) OR (Illness, Critical) OR (Illnesses, Critical) OR (Critically Ill) | 15727 |
| #9 | #7 OR #8 | 15727 |
| #10 | MeSH descriptor: [Placebos] explode all trees | 26719 |
| #11 | #3 OR #6 OR #10 | 34551 |
| #12 | MeSH descriptor: [Enteral Nutrition] explode all trees | 2555 |
| #13 | (Enteral Nutrition) OR (Tube Feeding) OR (Feeding, Tube) OR (Enteral Feeding) OR (Feeding, Enteral) OR (Gastric Feeding Tube) OR (Feeding Tube, Gastric) OR (Feeding Tubes, Gastric) OR (Gastric Feeding Tubes) OR (Gastric Feeding Tube) OR (Tube, Gastric Feeding) OR (Tubes, Gastric Feeding) OR (Force Feeding) OR (Feeding, Force) OR (Force Feedings) OR (Feedings, Force) | 10492 |
| #14 | #12 OR #13 | 10492 |
| #15 | #9 AND #11 AND #14 | 84 |

**Search strategy of web of science**

| NO. | Search deatiles | Results |
| --- | --- | --- |
| 1 | Erythromycin (Topic) | 25774 |
| 2 | (((((((((((Erythromycin A) OR (Erythromycin Lactate)) OR (Lactate, Erythromycin)) OR (Erythromycin Phosphate)) OR (Phosphate, Erythromycin)) OR (Erythromycin C)) OR (Ilotycin)) OR (Erycette)) OR (Erymax)) OR (T-Stat)) OR (TStat)) OR (T Stat) (Topic) (Topic) | 22871 |
| 3 | #1 OR #2 | 25970 |
| 4 | Metoclopramide (Topic) | 6003 |
| 5 | (((((((((((((Metoclopramide) OR (4-Amino-5-chloro-N-(2-(diethylamino)ethyl)-2-methoxybenzamide)) OR (Reglan)) OR (Primperan)) OR (Rimetin)) OR (Maxolon)) OR (Dihydrochloride)) OR (Dihydrochloride, Metoclopramide)) OR (Metoclopramide Hydrochloride)) OR (Hydrochloride, Metoclopramide)) OR (Metoclopramide Monohydrochloride, Monohydrate)) OR (Metoclopramide Monohydrochloride)) OR (Monohydrochloride, Metoclopramide)) OR (Cerucal) (Topic) (Topic) | 7280 |
| 6 | #4 OR #5 | 12936 |
| 7 | Placebos (Topic) | 323102 |
| 8 | #3 OR #6 OR #7 | 359965 |
| 9 | Critical Illness (Topic) | 43092 |
| 10 | (((Critical Illnesses) OR (Illness, Critical)) OR (Illnesses, Critical)) OR (Critically Ill) (Topic) | 131686 |
| 11 | #9 OR #10 | 131686 |
| 12 | Enteral Nutrition (Topic) | 20846 |
| 14 | #8 AND #11 AND #12 | 321 |

**Search strategy for the Chinese Databases:**

**Search strategy of the China National Knowledge Infrastructure (CNKI)：**

(SU='甲氧氯普胺' OR SU='胃复安' OR SU='灭吐灵' OR SU='Metoclopramide' OR SU='红霉素' OR SU='Erythromycin' OR SU='安慰剂' OR SU='Placebo' OR SU='空白对照' OR SU='假药') AND (SU='危重症' OR SU='危重病' OR SU='重症' OR SU='严重' OR SU='ICU' OR SU='加强医疗病房' OR SU='急诊' OR SU='抢救') AND (SU='肠内营养' OR SU='EN' OR SU='enteral nutrition')

**Search strategy of the Wanfang** **Database：**

（（主题:("甲氧氯普胺" or "胃复安" or "灭吐灵" or "Metoclopramide") * or 主题:("红霉素" or "Erythromycin") * or 主题：(“安慰剂” or “Placebo” or “空白对照” or “假药”)） and 主题:("危重病" or "危重症" or "重症" or "ICU" or "加强医疗病房" or "急诊" or "抢救") and 主题:(”肠内营养“ ) ) ）

**Search strategy of the VIP Database：**

(U='甲氧氯普胺' OR U='胃复安' OR U='灭吐灵' OR U='Metoclopramide' OR U='红霉素' OR U='Erythromycin' OR U='安慰剂' OR U='Placebo' OR U='空白对照' OR U='假药') AND (U='危重症' OR U='危重病' OR U='重症' OR U='严重' OR U='ICU' OR U='加强医疗病房' OR U='急诊' OR U='抢救')
