# Supplementary material for: Efficacy and safety of erythromycin and metoclopramide, alone or in combination, for successful feeding in critically Ill patients: a systematic review and network meta-analysis
Source: Front Nutr. 2026 Sep 9;13:1834838. doi: 10.3389/fnut.2026.1834838 (PMC13597321; doi:10.3389/fnut.2026.1834838)
Supplement: Supplementary file 3 [file Supplementary_file_3.docx]

**Supplementary Table 1. English versions of abstracts and URL from the included non-English published studies**

| **Study** | **URL** | **Abstract in English** |
| --- | --- | --- |
| Zhang 2015 | https://qikan.cqvip.com/Qikan/Article/Detail?id=664010719 | Objective: To evaluate the clinical efficacy of high-dose naloxone in treating gastric emptying disorders in critically ill patients.  Methods: A total of 385 patients requiring early enteral nutrition support were enrolled. After excluding 61 cases with clinical deterioration, 324 patients were divided into four groups: Metoclopramide Group (76 cases, 10 mg intravenously every 8 hours); Erythromycin Group (83 cases, 0. 25g intravenous infusion every 12 hours); Metoclopramide-Erythromycin Combination Therapy Group (84 cases, same regimen); and High-dose Naloxone Group (81 cases, 5 mg intravenous infusion for 1 hour every 8 hours). All patients received medication at 8:00 AM on the first day after ICU admission. Daily gastric retention volumes and gastrointestinal feeding status were recorded for all groups.  Results: ① The gastric juice retention volume in the combined therapy group from day 1 to 7 was significantly lower than that in the metoclopramide group and erythromycin group (P <0.01, P <0.05). From day 3 to 7, the high-dose naloxone group showed significantly lower gastric juice retention compared to both metoclopramide and erythromycin groups (P <0.05, P <0.01), but no significant difference was observed when compared with the combined therapy group (P> 0.05). ② The feeding success rate in the combined therapy group was significantly higher than in the metoclopramide and erythromycin groups after day 3 (P <0.01). The high-dose naloxone group demonstrated significantly higher feeding success rates than the other two groups after day 4 (all P <0.01), though no significant difference was found when compared with the combined therapy group (P> 0.05). Conclusion: High-dose naloxone exhibits effective gastrointestinal motility promotion. Although its onset is gradual, the effects are prolonged and pronounced. |
| Lu 2010 | https://pubmed.ncbi.nlm.nih.gov/20092709/ | **Objective:** To compare the effect of erythromycin and metoclopramide on feeding intolerance of critically ill patients in intensive care unit (ICU).  **Methods:** One hundred and fifty-two critically ill patients in ICU who needed early enteral nutrition exceeding 7 days between January 2007 and January 2009 were included in the study. The patients were randomly divided into three groups: erythromycin group (200 mg intravenous drip, once every 12 hours), metoclopramide group (10 mg intravenous injection, once every 8 hours), and combination therapy group. The whole experiment was carried out for 7 days. Residual gastric volume was aspirated and measured every day at 03:00, 09:00, 15:00 and 21:00. The daily mean gastric residual volume was compared. At the same time, the daily effectiveness of erythromycin and metoclopramide on the success of feeding was also compared. The factors associated with a poor response to prokinetic therapy were looked for.  **Results:** The daily gastric residual volume in the combination therapy group was smallest, the maximum was (40+/-8) ml; the maximum of gastric residual volume in erythromycin group was (42+/-7) ml; the maximum of gastric residual volume in metoclopramide group was (59+/-8) ml (P<0.05 or P<0.01). The successful rate of feeding was highest in the combination therapy group, and it was as high as 97.4%, the erythromycin group ranked the second (90.0%), and that of the metoclopramide group was lowest (89.5%, P<0.05 or P<0.01). Factors that were associated with a poor response to prokinetic therapy was high pretreatment 24-hour gastric residual volume (r=-0.584, P=0.000), high blood sugar level (r=-0.345, P=0.029), a high acute physiology and chronic health evaluation II (APACHEII) score (r=-0.437, P=0.005), and requirement for inotropic drug support (r=-0.389, P=0.041).  **Conclusion:** Low dose of erythromycin could improve the successful rate of feeding in critically ill patients in ICU. The combined administration of erythromycin and metoclopramide was more effective. Its side effect was minimal. |
| Chen 2011 | https://kns.cnki.net/kcms2/article/abstract?v=xhAGsX4BfX7g4Z2SbxqhCMt1M-QxF_ujQeqKDdxokVYFVmErV5dkBHLV_oiD2sG3BtKgusTsvadfmkQmylpQPd_MAlM9K92FB0WNKL1gE-KiS6RUrciYMBNpBmGpKzmGtpaowVbi54QBrO_bOM225HjjrU_tWjeQo6wkOMtZBJ0eDtVXaKbocC8_NMRUmFff&uniplatform=NZKPT&language=CHS | **Objective:**To investigate whether erythromycin and metoclopramide can improve the success rate of postpyloric intubation with spiral nasogastric tubes.  **Methods:** A total of 120 critically ill patients requiring enteral nutrition admitted to the Department of Critical Care Medicine at Guangdong Provincial People's Hospital from July 2006 to March 2007 were randomly divided into three groups: Erythromycin Group, Metoclopramide Group, and Control Group. The Erythromycin Group received 250mg lactobasic erythromycin intravenously for 12h before intubation, followed by repeated administration. The Metoclopramide Group received 10mg metoclopramide intravenously for 12h before intubation, followed by repeated administration. The Control Group received no gastrointestinal motility drugs. After 24h of tube placement, bedside abdominal X-ray was performed to confirm tube positioning.  **Results:** The postpyloric intubation success rates were 74.3% (29/39) in the Erythromycin Group and 71.8% (28/39) in the Metoclopramide Group, significantly higher than the 42.8% (18/42) in the Control Group (all P <0.05).  **Conclusion:** Erythromycin and metoclopramide can improve the success rate of postpyloric intubation with spiral nasogastric tubes. |
| Feng 2019 | <https://link.cnki.net/doi/10.16281/j.cnki.jocml.2019.63.148> | **Objective:** To investigate the impact of erythromycin combined with metoclopramide on the success rate of manual nasogastric tube placement.  **Methods:** This study included 68 critically ill patients admitted to our hospital between December 2017 and December 2018. Patients were randomly divided into two groups (34 cases each) using a dice roll method. The control group received erythromycin alone during manual tube placement, while the study group additionally used metoclopramide. Comparative analysis was conducted to evaluate tube placement outcomes.  **Results:** The study group achieved a 91.18% success rate versus 70.59% in the control group (P<0.05), demonstrating statistically significant differences. The study group also showed 2.94% complication incidence compared to 17.65% in the control group (P<0.05), with statistically significant variations.  **Conclusion:** The combined use of erythromycin and metoclopramide in manual nasogastric tube placement significantly improves success rates and reduces complications, making this approach highly recommended for clinical implementation. |
| Liu 2015 | <https://d.wanfangdata.com.cn/thesis/CiBUaGVzaXNOZXdTMjAyNTA2MTMyMDI1MDYxMzE2MTkxNhIHRDc3ODc4NxoIMjVkMzlib2w%3D> | **Objective:** To investigate the effects of erythromycin on gastric emptying disorders in critically ill patients through comparative analysis of erythromycin, metoclopramide, and their combination therapy.  **Methods:** Patients diagnosed with gastric retention and meeting the criteria for enteral nutrition (prolonged enteral feeding>7 days) were enrolled between October 2013 and October 2014 at the Department of Critical Care Medicine, Affiliated Hospital of North China University of Science and Technology. Exclusion criteria included: ① Contraindications to enteral nutrition (e.g., abdominal surgery within 6 weeks, major gastrointestinal bleeding, acute pancreatitis, mechanical biliary obstruction, suspected intestinal obstruction or perforation); ② Anatomical abnormalities (e.g., prior esophageal or gastrectomy); ③ Age <18 years; ④ Concurrent use of erythromycin, metoclopramide, domperidone, mosapride, or similar medications within 72 hours prior to enrollment; ⑤ Myasthenia gravis; ⑥ Pregnancy; ⑦ Gastric bleeding or esophageal varices without prior use of H2 receptor antagonists or proton pump inhibitors; ⑧ Concurrent use of drugs interacting with erythromycin or metoclopramide (e.g., atropine, scopolamine, ergotamine tartrate, digoxin, theophylline, warfarin, phenothiazine antidepressants, bromocriptine, carbamazepine, phenytoin sodium, valproic acid); ⑨ Elevated liver function enzymes (ALT, AST, LDH ≥3 times normal); ⑩ Allergy to macrolide antibiotics; ⑪ Patient or legal guardian refusal to participate. Patients received either erythromycin 250mg or metoclopramide 10 mg intravenously twice daily. Gastric retention volume was assessed twice daily. Feeding failure was defined as two or more episodes of ≥ 250ml gastric retention within the first 24 hours, or one episode of ≥250 ml within 24 hours. Patients with feeding failure underwent corrective treatment using combination therapy administered as in monotherapy. Clinical parameters monitored included: patient age, gender, BMI, APACHE II score; diagnostic indicators (blood glucose, plasma albumin, medications, mechanical ventilation mode); gastric retention volume changes, treatment efficacy, blood glucose, ALT, AST, ALB, GGT levels, infection rates, average hospital stay, ICU duration, and adverse events. All data were managed in Excel and analyzed using SPSS 17.0. Continuous variables were expressed as mean±SD or median (quartiles) depending on normal distribution, while categorical variables were presented as cases (%). Paired t-tests were used for normally distributed continuous variables, Mann-Whitney U test for skewed distributions, and χ² test for 2×2 or R×C contingency tables. Logistic regression was employed to analyze correlations. Statistical significance was defined as P<0.05.  **Results:** This trial enrolled a total of 131 patients, with 11 dropping out due to family decision to discontinue treatment, death, or hospital transfer. Ultimately, 120 patients completed the trial, divided equally between erythromycin (60 cases) and metoclopramide (60 cases) groups. After intervention, 30 patients in the erythromycin group and 37 in the metoclopramide group experienced feeding failure. All remaining patients were assigned to the remedial treatment group, with 13 additional dropouts during observation. Ultimately, 51 patients completed the remedial treatment process. Thirteen patients still had unresolved gastric retention after treatment, with 10 opting for jejunal nutrition tube placement during gastroscopy. Erythromycin group showed superior gastric retention resolution within 7 days (days 1-4) compared to metoclopramide group (P<0.05). No significant differences were observed in blood glucose, alanine aminotransferase (ALT), aspartate aminotransferase (AST), alkaline phosphatase (ALP), or γ-glutamyl transferase (GGT) levels during intervention (P>0.05). Statistical significance was noted in infection rates (8 vs. 21 cases, P=0.01), average hospitalization duration (17.3±3.2d vs. 25.1±5.6d, P<0.001), and ICU stay time (9.2±2.1d vs. 14.8±4.3d, P<0.001) between successful and failed feeding patients. Statistically significant differences were observed in average hospitalization duration (18.5±2.7d vs. 23.9±4.8d, P<0.001) and ICU stay (10.8±2.6d vs. 14.5±3.9d, P<0.001) between patients with successful and unsuccessful metoclopramide feeding. Significant differences in treatment efficacy were noted between the erythromycin group and metoclopramide group during days 1-4 (P<0.05), while marked variations were observed between the erythromycin group and the combined therapy group during days 5-7 (P<0.05). Cumulative adverse reactions occurred in 12 cases in the erythromycin group, 8 in the metoclopramide group, and 5 in the combined therapy group. Analysis of factors affecting treatment efficacy revealed that pre-treatment gastric residual volume was an independent risk factor (P=0.01).  **Conclusions:** 1. Erythromycin demonstrates good efficacy and safety when administered intravenously for treating gastric retention in critically ill patients, with early intervention showing significant benefits. 2. The combination of erythromycin and metoclopramide serves as a safe and effective salvage therapy for patients unresponsive to monotherapy, offering prolonged therapeutic effects. |

**Supplementary Table 2. Distribution of potential effect modifiers across included studies**

| **Study** | **Population** | **Age (years, mean/range)** | **Severity score (APACHE II or equivalent)** | **Erythromycin dose (monotherapy/combination)** | **Metoclopramide dose (monotherapy/combination)** | **Definition of feeding success (GRV threshold)** | **Definition of postpyloric intubation success** | **Concomitant medications (sedatives/opioids/vasopressors)** |
| --- | --- | --- | --- | --- | --- | --- | --- | --- |
| Chen 2011 | Adults | 65.9–72.1 | APACHE II 21.2–21.9 | ERY 250 mg x2 (IV) | MET 10 mg x2 (IV) | Not directly reported (primary outcome was tube placement) | Tip beyond pylorus (X‑ray) | Use of GI motility‑impairing drugs (sedatives, analgesics, muscle relaxants, β‑agonists): 10–17/39-42 |
| Dickerson 2009 | Adults (trauma) | 40–41 | ISS 32–36 | Only combination: ERY 250 mg q6h | MET 10 mg →20 mg q6h →combination | GRV >200 mL or vomiting/abdominal distension (for intolerance) | N/A | Most patients received sedatives/opioids (not quantified) |
| Feng 2019 | Adults | 52.0±6.2 | APACHE II 21.2±5.3 | ERY 0.5 mg/kg x2 | Combination: MET 10 mg x2 | N/A | Tip beyond pylorus (X‑ray) | Not reported |
| Gharpure 2001 | Children | 1.8–3.5 years | PRISM 5 (0–21) | ERY 10 mg/kg | — | N/A | Tip at or beyond third part of duodenum | High use of morphine, benzodiazepines, barbiturates |
| Griffith 2003 | Adults | 54.6–59.7 | Not reported (all mechanically ventilated) | ERY 500 mg single dose | — | N/A | Tip at or beyond second part of duodenum | All patients sedated, mechanically ventilated |
| Heiselman 1995 | Adults | Not reported | Not reported | — | MET 10 mg single dose | N/A | Radiologically confirmed transpyloric (X‑ray) | Patients receiving GI‑affecting drugs were excluded |
| Hu 2018 | Adults | 55.7–58.7 | APACHE II 21 (16–25) | ERY 500 mg q6h x4 | MET 20 mg (or 10 mg if renal impairment) | N/A | Tip at or beyond first part of duodenum (X‑ray) | Sedatives (32–37%), vasopressors (19–22%) |
| Ketsuwan 2021 | Children | 1.5–4.8 (median) | PRISM III 8 (7) | — | MET 0.1 mg/kg | N/A | Tip in duodenum or jejunum (X‑ray) | Sedatives/anaesthetics (48–65%) |
| Liang 2025 | Adults | 67 (53–76) | APACHE II 22 (18–27) | ERY 250 mg q6h x4 | MET 10 mg (weight‑adjusted, repeated) | N/A | Tip at or beyond first part of duodenum (X‑ray) | Sedatives (86–91%), vasopressors (34–40%) |
| Liu 2015 | Adults | 65–67 | APACHE II 22–23 | ERY 250 mg x2 (IV) | MET 10 mg x2 (IV) | GRV ≥250 mL defined as feeding failure | N/A | Opioids (43–45%), benzodiazepines (70–80%), catecholamines (65%) |
| Lu 2010 | Adults | Not reported | Not reported | ERY 200 mg q12h | MET 10 mg q8h | Not explicitly reported | N/A | Not reported |
| Maclaren 2008 | Adults | 48–51 | APACHE III 75–78 | ERY 250 mg q6h x4 | MET 10 mg q6h x4 | GRV ≥150 mL defined as intolerance | N/A | IV opioids (80–90%), vasopressors (10–20%) |
| Nguyen 2007A | Adults | 50–52 | APACHE II 26–27 | ERY 200 mg x2 (IV) | Combination: MET 10 mg x4 | GRV <250 mL and feeding rate ≥40 mL/h | N/A | Opioids+benzodiazepines (74–75%), vasopressors (56–63%) |
| Nguyen 2007B | Adults | 46–52 | APACHE II 24–26 | ERY 200 mg x2 (IV) | MET 10 mg x4 | GRV <250 mL and feeding rate ≥40 mL/h | N/A | Opioids+benzodiazepines (71–84%), vasopressors (44–47%) |
| Paz 1996 | Adults | 57–68 | Not reported | ERY 200 mg single dose | MET 10 mg single dose | N/A | Tip in duodenum (X‑ray at 24 h) | Morphine use (5–20%), but not systematically reported |
| Vijayaraghavan 2022 | Adults (cirrhosis) | 45–46 | SOFA 13.2–13.5 | ERY 70 mg q12h | MET 10 mg q8h | ≥3 of 5 criteria including GRV ≥500 mL/24h | N/A | Fentanyl (37–46%), propofol (48–54%) |
| Zhang 2015 | Adults | 57–68 | APACHE II 12–14 (low) | ERY 0.25 g q12h | MET 10 mg q8h | GRV ≤250 mL defined as success | N/A | Not reported |

**Notes:**

“—” indicates the intervention was not used or the outcome was not applicable.

“N/A” indicates the outcome was not reported or not relevant for that study.

GRV: gastric residual volume; IV: intravenous; q6h/q8h/q12h: every 6/8/12 hours; ISS: Injury Severity Score; SOFA: Sequential Organ Failure Assessment; PRISM: Pediatric Risk of Mortality.

The definitions of “feeding success” and “postpyloric intubation success” are provided as reported in each original study.

Concomitant medication use is listed only when explicitly reported; absence of reporting does not necessarily indicate absence of use.

**Supplementary Table 3. Sensitivity analysis for postpyloric intubation success after excluding pediatric studies (Gharpure 2001 and Ketsuwan 2021): SUCRA and PrBest rankings**

| **Treatment** | **SUCRA (%)** | **PrBest (%)** | **MeanRank** |
| --- | --- | --- | --- |
| combine | 77.2 | 54.8 | 1.9 |
| ERY | 52.1 | 5.0 | 2.9 |
| blankcontrol | 47.8 | 24.0 | 3.1 |
| metoclopramide | 46.6 | 11.0 | 3.1 |
| placebo | 26.2 | 5.2 | 4.0 |

**Supplementary Table 4. Sensitivity analysis for any reported adverse events after excluding pediatric studies (Gharpure 2001 and Ketsuwan 2021): SUCRA and PrBest rankings**

| **Treatment** | **SUCRA (%)** | **PrBest (%)** | **MeanRank** |
| --- | --- | --- | --- |
| metoclopramide | 74 | 23 | 2 |
| blank control | 66.7 | 46.6 | 2.3 |
| placebo | 50.9 | 30.1 | 3 |
| ERY | 43.8 | 0.3 | 3.2 |
| combine | 14.6 | 0 | 4.4 |

**Supplementary Table 5. Summary of any adverse events (AEs) from individual studies**

| **Study** | **Treatment** | **Any AE (n/N)** | **Digestive system** | **Liver enzyme elevation** | **Cardiovascular** | **Nervous system** | **Allergy/rash** | **Other (specified)** |
| --- | --- | --- | --- | --- | --- | --- | --- | --- |
| Chen 2011 | ERY | 1/39 | 0 | 0 | 0 | 0 | 0 | Phlebitis (1) |
|  | MET | 1/39 | 0 | 0 | 0 | Agitation (1) | 0 | 0 |
|  | BLANK | 0/42 | 0 | 0 | 0 | 0 | 0 | 0 |
| Feng 2019 | ERY | 6/34 | GI perforation (1), tube malposition (2), bleeding (3)* | 0 | 0 | 0 | 0 | 0 |
|  | COMB | 1/34 | Bleeding (1) | 0 | 0 | 0 | 0 | 0 |
| Gharpure 2001 | ERY | 5/37 | Vomiting (3), loose stool (1) | 0 | 0 | 0 | Rash (1) | 0 |
|  | PLACEBO | 1/37 | Vomiting (1) | 0 | 0 | 0 | 0 | 0 |
| Griffith 2003 | ERY | 0/14 | 0 | 0 | 0 | 0 | 0 | 0 |
|  | PLACEBO | 0/22 | 0 | 0 | 0 | 0 | 0 | 0 |
| Heiselman 1995 | MET | 0/59 | 0 | 0 | 0 | 0 | 0 | 0 |
|  | BLANK | 0/46 | 0 | 0 | 0 | 0 | 0 | 0 |
| Hu 2018 | ERY | 54/167 | Diarrhea (10), nausea (2), vomiting (2) | Liver dysfunction (9) | Arrhythmia (1) | 0 | Rash (1) | Asthenia (1), jaundice (1); tube‑related complications (10)** |
|  | MET | 45/165 | Not specified separately; drug‑related AEs reported in 4 patients but details not given; includes new IAIs and tube complications | 0 | 0 | 0 | Rash (1) | Tube complications (10), new ICU‑acquired infections (35) |
| Ketsuwan 2021 | MET | 0/42 | 0 | 0 | 0 | 0 | 0 | 0 |
|  | PLACEBO | 0/40 | 0 | 0 | 0 | 0 | 0 | 0 |
| Liang 2025 | ERY | 39/260 | Diarrhea (12), nausea (2), vomiting (2) | ALT/AST elevation (6) | Arrhythmia (1) | 0 | 0 | Tube complications (2), new IAIs (14) |
|  | MET | 26/259 | Diarrhea (3), nausea (2), vomiting (1) | 0 | Arrhythmia (1) | 0 | Rash (2) | Tube complications (3), new IAIs (12) |
|  | COMB | 48/256 | Diarrhea (13), nausea (3) | ALT/AST elevation (11) | Arrhythmia (2) | 0 | Rash (1) | Tube complications (3), new IAIs (15) |
| Liu 2015 | ERY | 12/60 | Digestive symptoms (7)† | AST elevation (5) | 0 | 0 | Allergic reaction (4) | Others (6)‡ |
|  | MET | 8/60 | Digestive symptoms (4) | 0 | 0 | Muscle twitching? | 0 | Dizziness, headache, drowsiness (6) |
|  | COMB†† | 5/51 | Digestive symptoms (4) | ALP elevation (3), AST elevation (2) | 0 | 0 | 0 | 0 |
| MacLaren 2008 | ERY | 3/10 | Diarrhea (3) | 0 | 0 | 0 | 0 | 0 |
|  | MET | 1/10 | Diarrhea (1) | 0 | 0 | 0 | 0 | 0 |
| Nguyen 2007A | COMB | 20/37 | Diarrhea (20) | 0 | 0 | 0 | 0 | 0 |
|  | ERY | 10/38 | Diarrhea (10) | 0 | 0 | 0 | 0 | 0 |
| Paz 1996 | ERY | 0/21 | 0 | 0 | 0 | 0 | 0 | 0 |
|  | MET | 0/20 | 0 | 0 | 0 | 0 | 0 | 0 |
|  | PLACEBO | 0/16 | 0 | 0 | 0 | 0 | 0 | 0 |
| Dickerson 2009§ | MET/COMB | – | Diarrhea reported (12% on metoclopramide 10 mg, 22% on 20 mg, 26% on combination) but no absolute numbers | 0 | 0 | 0 | 0 | 0 |

**Notes:**

* In Feng 2019, “GI perforation, tube malposition, bleeding” may be partly procedure‑related but were counted as AEs by the study authors.

** Tube‑related complications (e.g., airway misplacement, nasal mucosa bleeding, tube buckling) were not drug‑related but are included in the “any AE” counts as reported.

† Digestive symptoms in Liu 2015 include nausea, vomiting, diarrhea, abdominal pain; individual numbers not provided.

‡ Others in Liu 2015 (erythromycin group) include complex infection, arrhythmia, phlebitis (counted as 6 events but may overlap with patients).

§ Dickerson 2009 is a non‑randomized study; only percentages without absolute event numbers were reported, so it was not included in the quantitative NMA.

†† Liu 2015 combination arm was not a parallel randomised group (used as rescue therapy after monotherapy failure); its data are presented for qualitative completeness only and were excluded from the quantitative network meta‑analysis for any AEs.

The following studies had no mention of safety outcomes and were excluded from this table: Lu 2010, Nguyen 2007B, Paz 1996, Vijayaraghavan 2022, Zhang 2015.

Due to lack of individual‑level overlap data, the sum of specific symptom counts may overestimate the actual number of patients affected. The primary safety analysis in the NMA uses the composite endpoint “any reported AE” to avoid double‑counting.

**Supplementary Table 6. GRADE evidence summary for the four outcomes**

| **Comparison** | **No. of studies** | **Network estimate OR (95% CI)** | **Risk of bias** | **Inconsistency** | **Indirectness** | **Imprecision** | **Publication bias** | **Certainty of evidence (GRADE)** |
| --- | --- | --- | --- | --- | --- | --- | --- | --- |
| **Feeding success** | | | | | | | | |
| combine vs. ERY | 5 | 3.03 (1.90, 4.83) | Serious concern | No concern | No concern | No concern | No concern | ⊕⊕⊕◯ Moderate |
| metoclopramide vs. ERY | 4 | 0.62 (0.41, 0.94) | Serious concern | No concern | No concern | No concern | No concern | ⊕⊕⊕◯ Moderate |
| metoclopramide vs. combine | 4 | 0.20 (0.13, 0.33) | Serious concern | No concern | No concern | No concern | No concern | ⊕⊕⊕◯ Moderate |
| **Postpyloric intubation success** | | | | | | | | |
| combine vs. ERY | 2 | 1.69 (0.31, 9.10) | Serious concern | Serious concern | No concern | Very serious concern | No concern | ⊕◯◯◯ Very low |
| metoclopramide vs. ERY | 5 | 1.00 (0.31, 3.24) | Serious concern | No concern | No concern | Very serious concern | No concern | ⊕◯◯◯ Very low |
| placebo vs. ERY | 4 | 0.41 (0.10, 1.69) | Serious concern | No concern | No concern | Very serious concern | No concern | ⊕◯◯◯ Very low |
| combine vs. blank control | 0 (indirect) | 2.38 (0.11, 51.5) | No concern | No concern | Serious concern | Very serious concern | No concern | ⊕◯◯◯ Very low |
| **GRV** | | | | | | | | |
| combine vs. ERY | 1 (direct) + indirect | -11.28 (-206.68, 184.12) | Serious concern | No concern | No concern | Very serious concern | No concern | ⊕◯◯◯ Very low |
| metoclopramide vs. ERY | 3 (direct) | -65.67 (-192.97, 61.63) | Serious concern | No concern | No concern | Very serious concern | No concern | ⊕◯◯◯ Very low |
| metoclopramide vs. combine | 1 (direct) + indirect | -54.39 (-249.86, 141.08) | Serious concern | No concern | No concern | Very serious concern | No concern | ⊕◯◯◯ Very low |
| **Any reported adverse events** | | | | | | | | |
| metoclopramide vs. ERY | 6 | 0.70 (0.51, 0.96) | Serious concern (-1) | No concern | No concern | No concern | No concern | ⊕⊕⊕◯ Moderate |
| placebo vs. ERY | 3 | 0.38 (0.08, 1.81) | Serious concern (-1) | No concern | No concern | Serious concern (-1) | No concern | ⊕⊕◯◯ Low |
| combine vs. ERY | 4 | 1.45 (0.99, 2.14) | Serious concern (-1) | No concern | No concern | Serious concern (-1) | No concern | ⊕⊕◯◯ Low |
| blank vs. ERY | 1 | 0.53 (0.05, 6.20) | Serious concern (-1) | No concern | No concern | Very serious concern (-2) | No concern | ⊕◯◯◯ Very low |

**NOTES：**

Explanation of symbols

“Serious concern”: the domain has important limitations, downgrade by one level.

“Very serious concern”: the domain has very important limitations, downgrade by two levels.

“No concern”: no downgrading for that domain.

“⊕⊕⊕⊕” High; “⊕⊕⊕◯” Moderate; “⊕⊕◯◯” Low; “⊕◯◯◯” Very low.

“–” indicates that no direct studies exist for that comparison; the network estimate is derived solely from indirect evidence.

Additional notes

For the GRV outcome, the effect measure is mean difference (MD) in mL; for all other outcomes, it is odds ratio (OR).

In the “No. of studies” column: for comparisons with direct evidence, the number of direct studies is shown; for comparisons without direct evidence (e.g., combine vs. blank control), “0 (indirect)” is indicated. For GRV, “1 (direct) + indirect” means there is one direct study and the network estimate also incorporates indirect evidence.

Upgrading factors (large effect, dose‑response, plausible confounding that would reduce the effect) were not applicable in this study and are therefore not listed.
